# Comparative RNA-Seq Analysis Uncovers a Complex Regulatory Network for Soybean Cyst Nematode Resistance in Wild Soybean (*Glycine soja*)

**DOI:** 10.1038/s41598-017-09945-0

**Published:** 2017-08-29

**Authors:** Hengyou Zhang, Susanne Kjemtrup-Lovelace, Changbao Li, Yan Luo, Lars P. Chen, Bao-Hua Song

**Affiliations:** 1 Department of Biological Sciences, University of North Carolina at Charlotte, Charlotte, NC 28223 USA; 2 Department of Plant and Microbial Biology, North Carolina State University, Raleigh, NC 27695 USA; 3 Double Haploid Optimization Group, Monsanto Company, St. Louis, MO 63167 USA; 4 Xishuangbanna Tropical Botanical Garden, Chinese Academy of Sciences, Yunnan 650221, China; 5 Biology Department, University of North Carolina, Chapel Hill, NC 27599 USA

## Abstract

Soybean cyst nematode (SCN) is the most damaging pest of soybean worldwide. The molecular mechanism of SCN resistance remains largely unknown. We conducted a global RNA-seq comparison between a resistant genotype (S54) and a susceptible genotype (S67) of *Glycine soja*, the wild progenitor of soybean, to understand its regulatory network in SCN defense. The number of differentially expressed genes (DEGs) in S54 (2,290) was much larger than that in S67 (555). A number of defense-related genes/pathways were significantly induced only in S54, while photosynthesis and several metabolic pathways were affected in both genotypes with SCN infection. These defense-associated DEGs were involved in pathogen recognition, calcium/calmodulin-mediated defense signaling, jasmonic acid (JA)/ethylene (ET) and sialic acid (SA)-involved signaling, the MAPK signaling cascade, and WRKY-involved transcriptional regulation. Our results revealed a comprehensive regulatory network involved in SCN resistance and provided insights into the complex molecular mechanisms of SCN resistance in wild soybean.

## Introduction

Soybean cyst nematode (SCN; *Heterodera glycines* Ichinohe) is the most damaging pest for soybean (*Glycine max* (L.) Merr.). The value of soybean lost to SCN was estimated at 1.5 billion dollars in the United States^1^. The nematode life cycle includes four juvenile stages and an adult stage^2^. The infective second-stage juvenile (J2) can penetrate through epidermal cells of the root and establish a permanent feeding site, also called a syncytium. Establishment of an *H. glycines* syncytium can occur in either resistant or susceptible soybean roots in the early invading stage. However, in contrast to the healthy growth of *H. glycines* during a compatible reaction in soybean roots, the growth and reproduction of *H. glycines* are significantly inhibited, and syncytial collapses are also observed soon after their formation during a resistance reaction in soybean roots^3, 4^.

In addition to the visible cellular changes after *H. glycines* invasion in soybean roots, several groups have used microarray-based global transcriptome analysis to characterize resistant and susceptible responses to various SCN populations (HG types) of *H. glycines* ^2–9^. Thus far, most of these transcriptome profiling studies have been focused on HG type 0 (race 3), the most prevalent HG type in the central United States. Detailed examinations of soybean responses to *H. glycines* were performed spatially and temporally. Most studies examined soybean responses within a period from 2 days post-inoculation (dpi) to 10 dpi, during which the formation of syncytia and syncytial collapses were observed in the context of resistance responses^3, 4^, suggesting the importance of this time period for resistant soybeans in defending against *H. glycines*. In addition, soybean reactions to *H. glycines* infection have been characterized by examining whole infected roots^7, 8^ and local feeding sites^3–6^. Consequently, a broad diversity of gene families related to defense resistance with highly induced gene expression has been associated with SCN resistance. These genes include nucleotide-biding site-leucine-rich repeat family (*NBS-LRR*) genes, heat shock protein genes (*HSPs*), WRKY transcription factors, pathogenesis-related genes (*PR*), phenylpropanoid metabolism genes, and ethylene metabolism genes. These gene-chip-based studies have increased our understanding of the mechanisms of soybean-nematode interactions.

Compared to the microarray-based transcriptome analyses, deep sequencing of RNA-seq-based analyses can enable researchers to generate an unprecedented global view of the transcriptome changes and to extract the signaling pathways responsible for plant defenses to various biotic stresses^10–12^. Recently, several studies have used RNA-seq assays to quantify changes in the soybean transcriptome upon *H. glycines* infection to HG type 0^13, 14^. Using RNA-seq analysis, Hosseini and Matthews^15^ revealed not only a number of candidate genes that were previously identified^3, 4, 6^ but also a small subset of novel defense-response candidate genes.

Most previous studies used cultivated soybeans to identify candidate genes involved in SCN resistance. However, the cultivated soybean has undergone genetic bottlenecks and has lost more than half of its genetic variation^16^, which is one of the critical challenges for further improvement of diverse soybean SCN resistance varieties. For example, most of the commercial resistance cultivars were derived from limited resistant sources, such as PI88788, Peking, and PI437654^4^. Overuse of these resistant cultivars resulted in *H. glycines* type shifts, and some of the PI88788-derived resistant soybean cultivars are losing resistance^17^. Thus, there is an urgent need to identify novel and diverse genetic sources resistant to *H. glycines*. We used wild soybean (*Glycine. soja* Sieb. & Zucc.), which harbors much higher genetic variation than cultivated soybean, as a study system to explore these untapped genetic resources for managing SCN damages. Long-term improvement of soybean production and uncovering of the molecular mechanisms of *H. glycines* resistance represent equally important benefits of *H. glycines* management. Recently, we have identified wild soybean ecotypes resistant to HG type 2.5.7, and new resistance mechanisms might exist in *G. soja* ^18^. To extend this study and to gain insights on the molecular mechanisms of SCN resistance in *G. soja*, we conducted an RNA-seq based transcriptome comparison between the resistant (S54) and susceptible (S67) genotypes of *G. soja*. We aimed to identify the key genes and/or important signaling pathways involved in wild soybean defense to HG type 2.5.7. In addition to plant defense genes previously identified using this approach, our research also uncovered some novel defense-response candidate genes. Further, biologically sound regulatory pathways and networks involved in *H. glycines* resistance were also proposed.

## Results

### Nematode growth was inhibited in resistant *G. soja* S54

**Figure 1:**
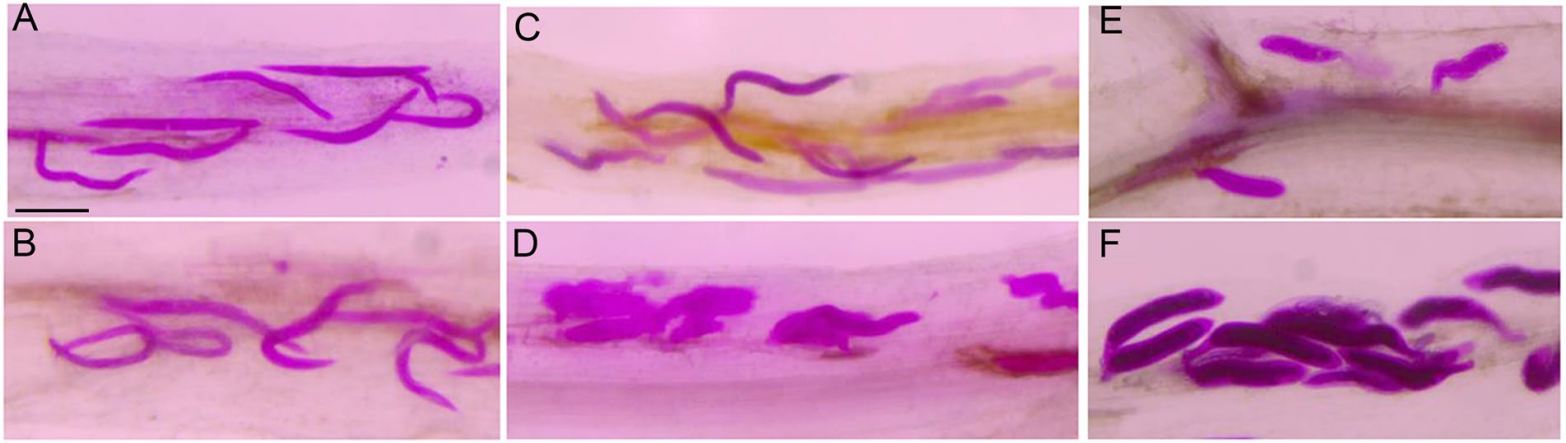
Penetration and development of HG type 2.5.7 in *G. soja* S54 (**A**,**C**,**E**) and S67 (**B**,**D**,**F**) roots. Roots with penetrated nematodes were acid fuschin-stained at different dpi. (**A**) S54 at 3 dpi. (**B**) S67 at 3 dpi. (**C**) S54 at 5 dpi. (**D**) S67 at 5 dpi. (**E**) S54 at 8 dpi. (**F**) S67 at 8 dpi. Bars = 250 μm.

The development of the nematode was investigated in both resistant and susceptible genotypes. The roots infected for 3, 5, and 8 dpi were isolated from plants and stained with acid fuchsin. We found similar sizes of *H. glycines* in both S54 and S67 at 3 dpi (Fig. 1A, B). By 5 dpi, significant differences in the development of nematodes were observed. In S67 roots, late third-stage juvenile (J3) nematodes were observed (Fig. 1D), whereas the nematodes did not grow to developmental stage J3 in S54 roots (Fig. 1C). By 8 dpi, the nematodes had advanced to late third or early forth-stage juveniles (J4) in S67 (Fig. 1F), while nematodes were in J3 stage in S54 (Fig. 1E). These results were consistent with the results from greenhouse screening assays, indicating that S54 was HG type 2.5.7-resistant.

### Transcriptome changes in S54 and S67 in response to HG type 2.5.7 infection

The Illumina sequencing generated a total of 244.6 million raw reads for twelve libraries, ranging from 16.1 and 26.3 million reads per library. After quality control, 99.2% to 99.6% of high-quality reads per library were saved (Table S1). All of the raw data were deposited in the NCBI’s Short Read Archive database under Accession Number SRRXXXXX. After aligning with TopHat, 85.5% to 93.0% of the reads per library were mapped to the *G. max* genome, with 80.7% to 87.9% uniquely mapped (Table S1). After analyses using Cufflinks, a total of 56,314 and 56,559 genes were found expressed in the S54 and S67 roots, respectively.

**Figure 2:**
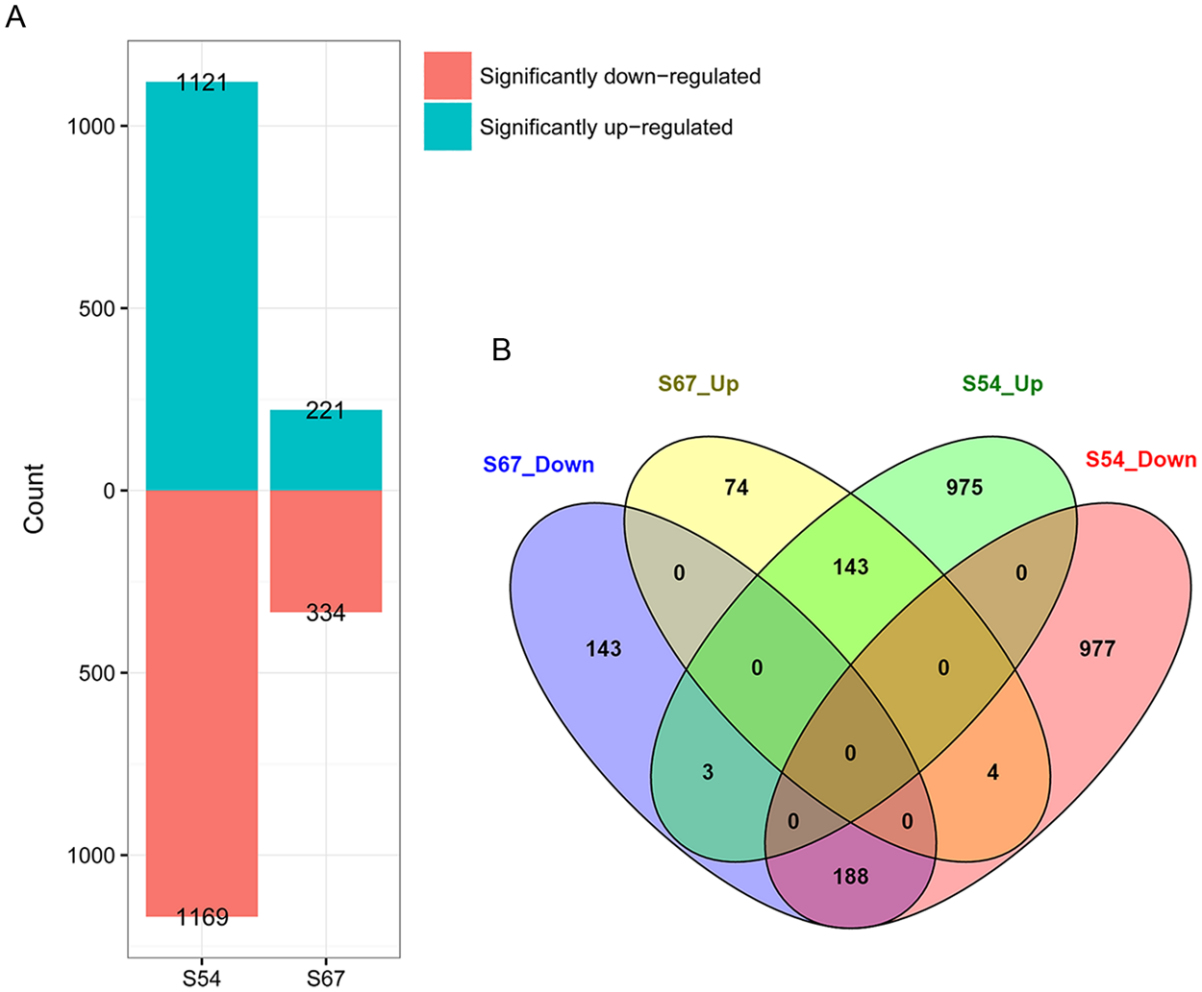
Differentially expressed genes (DEGs) of S54 and S67 in response to *H. glycines* infection. (**A**) Bar plot showing the numbers of up- and down-regulated DEGs in S54 and S67, respectively. (**B**) Venn diagram displaying the overlaps among different groups of DEGs.

To investigate the differential responses to HG type 2.5.7 infection between S54 and S67, we identified the DEGs between the treated and control roots for each genotype. In total, 2,290 genes were identified to be DEGs between treated and control roots in resistant S54, with 1,121 genes being significantly up-regulated and 1,169 genes being down-regulated (Fig. 2A, Table S2). In contrast, only 555 genes were differentially expressed in susceptible S67 upon *H. glycines* infection (Fig. 2A, Table S3). Volcano plots were used to visualize the results and are shown in Figure S1. The relationship between S54 and S67 DEG datasets was visualized in a Venn diagram (Fig. 2B). A majority of DEGs in S67 were also induced in S54 upon *H. glycines* infection. Briefly, S54 and S67 shared 143 significantly up-regulated and 188 significantly down-regulated DEGs, representing 64.7% and 56.3% of total significantly up- and down-regulated DEGs in S67.

**Figure 3:**
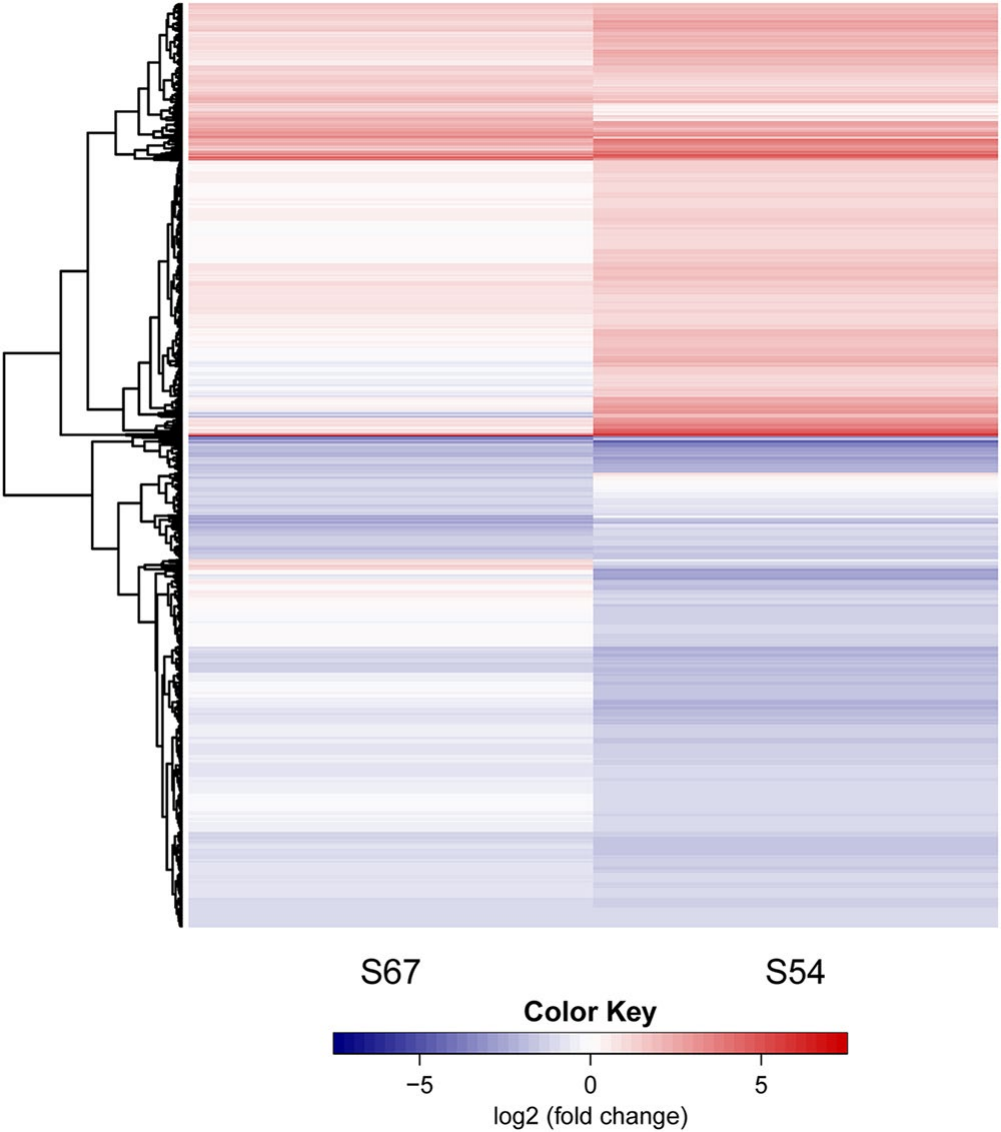
Heat map showing the expression patterns of all DEGs in S54 and S67.

To better understand the transcriptome changes to *H. glycines* infection in S54 and S67, a heatmap showing the expression patterns of all DEGs is shown in Fig. 3. A majority of these DEGs behaved similarly in both S54 and S67, with most of them showing more dramatic changes in S54 than in S67. However, the DEGs that were uniquely induced in S54 were nonresponsive to *H. glycines* infection in S67. A relatively small number of DEGs showed opposite expression profiles in both genotypes upon infection. Those S54-specific DEGs or the DEGs with more dramatic changes in expression in S54 than S67 might be significantly important in regulating cellular responses to defend against *H. glycines* attack.

To identify the important genes responsible for HG type 2.5.7 resistance, we extracted a subset of core DEG datasets for further analysis. Based on the Venn diagram shown in Fig. 3B, we chose the subset dataset consisting of the DEGs that were uniquely expressed in S54 (975 up-, 977 down-regulated), or exhibited opposite expression patterns between S54 and S67, and common DEGs whose fold change was more than 1.5-fold in S54 than that in S67. In total, 1,307 up- and 1,304 down-regulated DEGs in S54 were identified as the subset of DEGs used for the following analysis (Tables S4 and S5).

### GO and KEGG analyses revealed genes enriched in defense-related pathways

**Figure 4:**
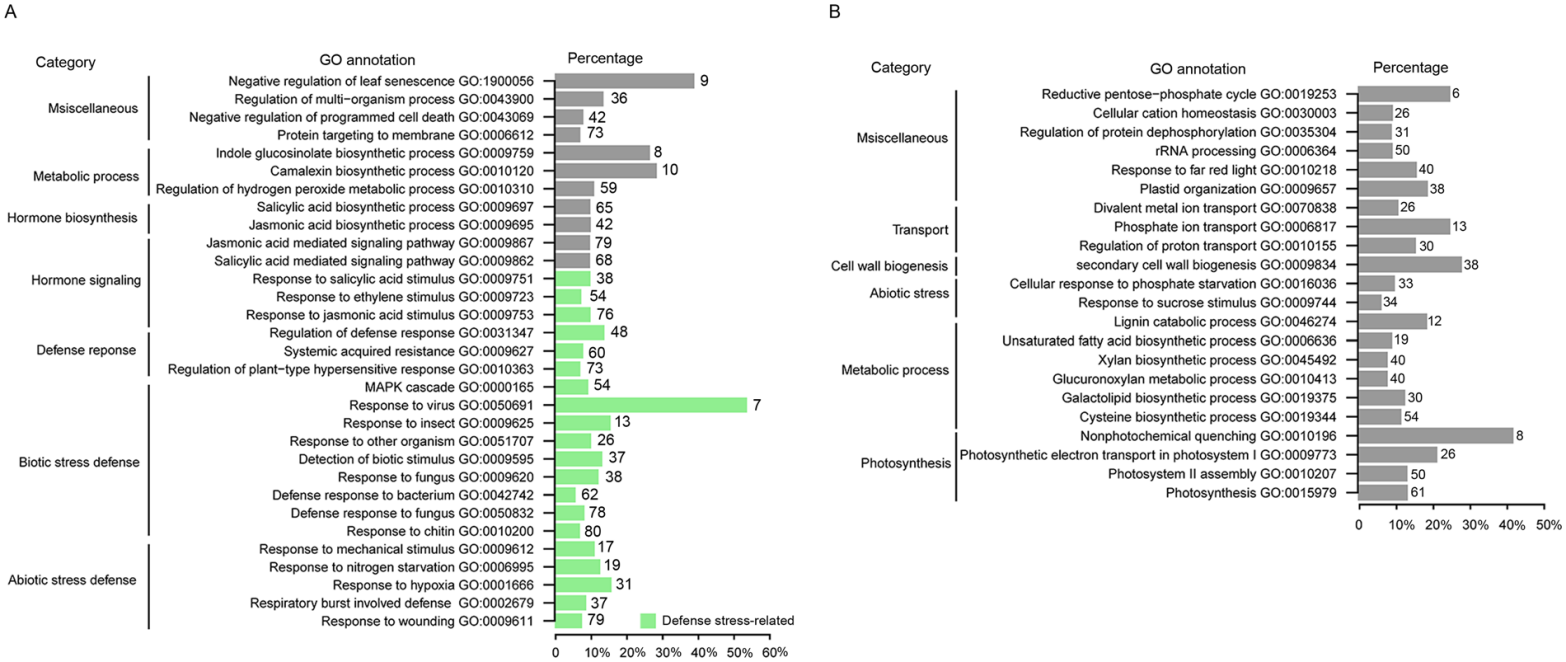
Gene Ontology (GO) enrichment analysis of up- (**A**) and down-regulated (**B**) core DEGs.

To gain insights into the biological processes associated with resistant responses to SCN HG type 2.5.7, we performed GO enrichment analysis to identify terms significantly over-represented for up- and down-regulated core DEGs (Fig. 4; Table S6). As shown in Fig. 4A, most of the enriched GO terms for up-regulated core DEGs belonged to three categories of biological processes, such as plant responses to abiotic stress and biotic stress, hormone signaling, and metabolic processes. Accordingly, GO terms related to three molecular functions, such as peroxidase activity, calcium ion binding, and kinase activity, were also overrepresented in up-regulated DEGs in S54 (Table S6). Interestingly, two secondary categories, including respiratory burst involved defense (GO:0002679) and response to chitin (GO:0010200), were enriched in both up-regulated DEGs in S54 (Fig. S3A; Table S15) and down-regulated DEGs in S67 (Fig. S3B). In contrast, two GO terms, syncytium formation (GO:006949) and plant-type cell wall loosening (GO:0009828), were enriched only in susceptible S67 (Fig. S3A). Differing from the enriched GO terms for up-regulated core DEGs, most categories of enriched GO terms for the down-regulated core DEGs dataset belonged to metabolic processes, photosynthesis, and developmental regulations (Fig. 4B; Table S6). These processes enriched in both genotypes might be independent of the regulatory network for *H. glycines* resistance and are instead affected by *H. glycines* infection in both S54 and S67. More enriched GO categories related to metabolic and photosynthesis processes were observed in S54 than in S67 (Fig. S3B).

To further obtain an overview of these biological processes altered during *H. glycines* infection, the core DEGs were assigned to KEGG pathways, as previously described^19^. In total, eight and six KEGG pathways were over-represented for up- and down-regulated DEGs, respectively (Table S6). Of particular note were two pathways, phenylpropanoid biosynthesis (gmx00940; Fig. S5A; Table S7) and plant-pathogen interaction (gmx04626; Fig. S5B; Table S7), which were enriched for up-regulated DEGs. In contrast, the photosynthesis-related pathways (gmx00195, gmx00196) were the top two enriched pathways for down-regulated DEGs, followed by metabolic pathways (gmx01100) and carbon fixation in photosynthetic organisms (gmx00710).

In addition, we also characterized the DEGs with the same responses between the two genotypes using GO and KEGG enrichment analysis (Fig. 2; Table S16). In the up-regulated DEGs, cellular response to ethylene stimulus and nitric oxide (GO terms), and biosynthesis of phenylpropanoid and secondary metabolites (KEGG terms), represent the top enriched terms, suggesting that these biological processes and pathways play important roles in the common defense response to *H. glycines* infection in the two genotypes. In the down-regulated DEGs in common between the two genotypes, photosynthesis pathway and biological processes involved in reduction-oxidation reactions represent the top enriched terms.

### Identification of important genes involved in HG type 2.5.7 resistance

**Figure 5:**
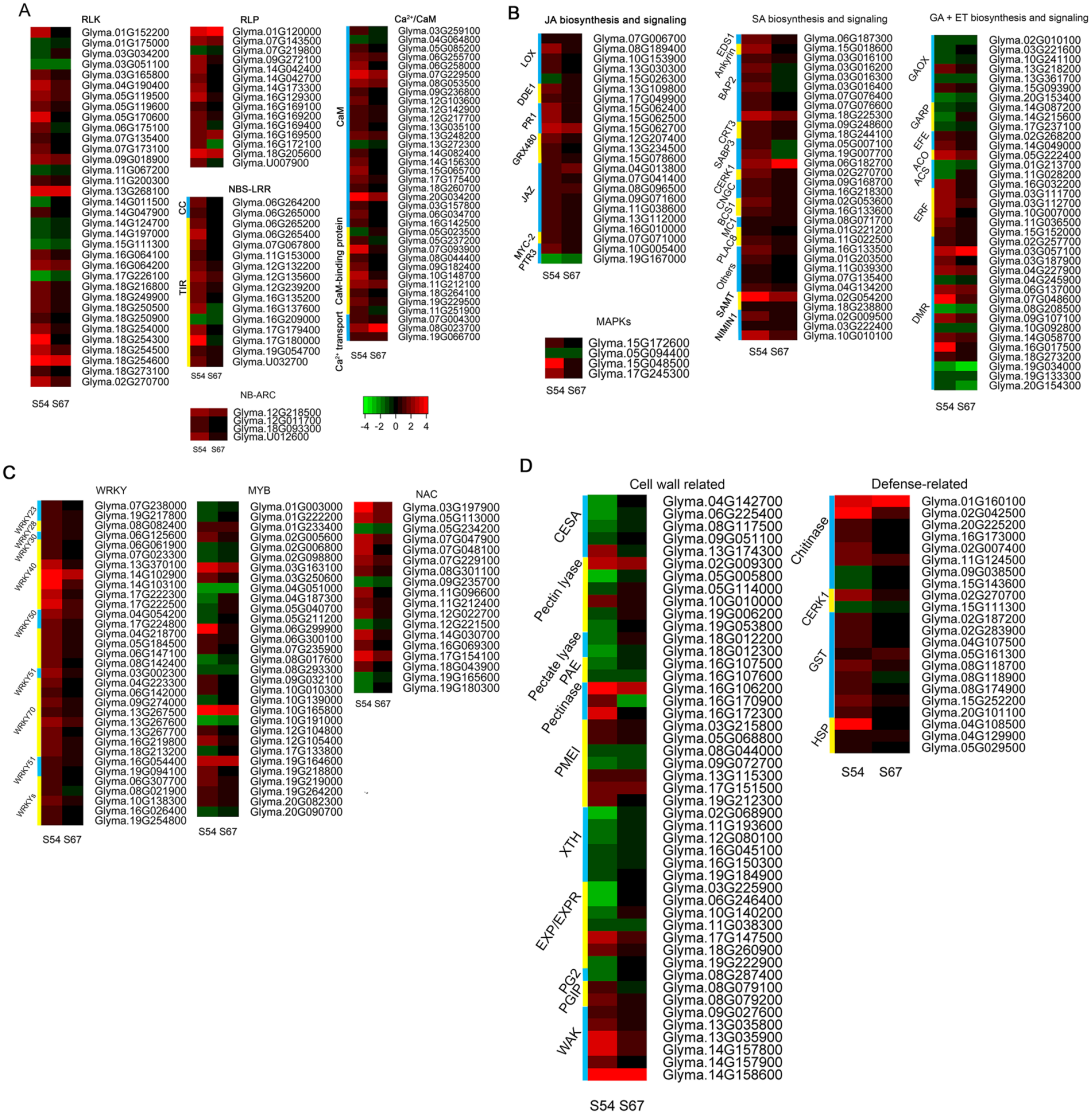
Heat map illustration of the representative DEGs involved in various defense-related pathways. (**A**) DEGs encoding RLK, RLP, NBS-LRR, NB-ARC, and calcium/calmodulin -related proteins. (**B**) DEGs involved in the biosynthesis of JA, SA/ET, and related signaling pathways. (**C**) DEGs encoding WRKY, MYB and NAC transcription factor families. (**D**) DEGs encoding cell wall-related proteins and other defense proteins.

Our results of comparative transcriptome analyses of responses to *H. glycines* HG type 2.5.7 between S54 and S67 indicated that soybean defense in response to *H. glycines* requires enrollment of a diversity of protein families involved in recognition of virulent proteins (Fig. 5A; Tables S8 and S9), signaling and post-translational modification by phosphorylation (Fig. 5A and B; Tables S8 and S9), calcium/calmodulin (Fig. 5A; Table S10), and phytohormone-mediated signaling (Fig. 5B; Table S10), transcriptional regulation by WRKY transcription factors (TFs) (Fig. 5C; Table S11), cell wall remodeling and associated signaling (Fig. 5D; Table S12), and various other defense-related proteins (Fig. 5D; Table S12).

### RLK, RLP and NBS-LRR proteins involved in the recognition of *H. glycines* secretions

Protein kinases play important roles in plant growth, development, and defense against various stresses^20^. In our study, a total of 121 protein kinases (PKs) were significantly induced by *H. glycines* infection, 70 (57.6%) of which were receptor-like protein kinases (RLKs; Table S9). These RLKs mainly included leucine-rich repeat (LRR)-RLKs, cysteine-rich RLKs, and wall-associated kinases. A majority of these kinases showed up-regulated expression in S54 while they were down-regulated or showed insensitive expression in S67 upon *H. glycines* infection (Table S9).

Here, we identified 27 LRR-RLKs as DEGs, with 22 showing up-regulated and five showing down-regulated expression in S54 upon *H. glycines* infection (Fig. 5A; Table S8). In contrast, eighteen of these 22 LRR-RLK DEGs showed insensitive expression in S67 following the infection. These 27 soybean LRR-RLK DEGs included several well-studied LRR-RLK homologs, such as two Brassinosteroid insensitive 1 (BRI1)-associated kinases (BAK1) (*Glyma.05G119500*, *Glyma.05G119600*)^21^ and two suppressors of BAK1-interacting receptor kinase 1 (*SOBIR1*) genes (*Glyma.04G190400*, *Glyma.06G175100*)^22, 23^. As for RLKs without LRR domains, two chitin elicitor receptor kinase 1 molecules (CERK1, *Glyma.02G270700*, *Glyma.15G111300*)^24^ that showed opposite expression patterns to each other in response to *H. glycines* infection were identified in S54 (Fig. 5A; Table S9). Other SCN-induced RLKs, such as two lectin receptor kinases (Glyma.03G051100, Glyma.07G135400), two proline-rich extensin-like receptor kinases (Glyma.01G175000, Glyma.11G067200), and six wall-associated kinases (WAKs), were also identified (Table S9). In addition, we also identified fifteen DEGs encoding receptor-like proteins (RLP), which might also be related to membrane-associated defense. Fifteen *RLPs* were induced, with ten showing up-regulated expression. Five of ten were significantly induced in S54 but not in S67 upon *H. glycines* infection (Fig. 5A; Table S8).

In addition to RLKs and RLPs that can serve as defense proteins involved in pathogen triggered immunity (PTI) to *H. glycines* infection, NBS-LRR family proteins involved in effector triggered immunity (ETI) were also identified as DEGs (Fig. 5A; Table S8). Here, we identified a total of sixteen *NBS-LRR* genes (5.02%) showing significantly enhanced expression levels following *H. glycines* attack, with one (*Glyma.16G209000*) showing down-regulated expression in S54. Of sixteen *NBS-LRR* genes, fourteen (87.5%) were in the TIR-NBS-LRR class, and two were CC-NBS-LRR members. Gene *Glyma.17G180000* was the most strongly induced among sixteen *NBS-LRR* DEGs with 12-fold up-regulation in S54 but showed insensitivity to HG type 2.5.7 infection in S67. Furthermore, four NB-ARC genes regulating the activity of the R proteins^25^ were significantly induced in S54 upon infection (Fig. 5A).

### Calcium/calmodulin-mediated signaling involved in *H. glycines* resistance

In our study, 23 genes encoding calmodulins (also known as Ca^2+^ sensor proteins or Ca^2+^-binding proteins) were identified to be DEGs, with 21 calmodulins being significantly induced in S54 upon *H. glycines* infection (Fig. 5A; Table S10). In contrast, eighteen of the 23 DEGs showed no significant change in expression levels in S67 upon infection.

In addition, genes involved in defense signaling downstream of Ca^2+^ sensor proteins (calmodulin) were also significantly induced by *H. glycines* infection (Fig. 5A; Table S10). Of the ten DEGs encoding calmodulin-binding proteins, nine of them showed significantly up-regulated expression in S54 upon *H. glycines* infection. Four (Glyma.05G237200, Glyma.07G093900, Glyma.08G044400, and Glyma.09G182400) of nine calmodulin-binding proteins were homologous to systemic acquired resistance deficient 1 (SARD1), which can constitutively activate a sialic acid (SA)-dependent defense response in *Arabidopsis* ^26^. A calmodulin-binding transcription activator (CAMTA)^27^ protein (*Glyma.11G251900*) showed significantly up-regulated expression in S54 but showed unchanged expression in S67 upon *H. glycines* infection. In addition, genes involved in Ca^2+^ transmembrane transport were also induced, such as autoinhibited Ca^2+^-ATPase 9 (ACA9, *Glyma.07G004300*) and protein calcium exchanger 7 (CAX7, *Glyma.19G066700*).

### MAPK cascades involved in defense signaling during *H. glycines* infection

MAPK cascade-mediated signaling plays remarkably important roles in plant defenses against a variety of biotic stresses^28^. The MAPK signaling cascades are minimally composed of a *MAPKKK*, a *MAPKK*, and a *MAPK* to link the upstream receptors to downstream targets. In this study, we identified a total of 36 *MAPKs*, 15 *MAPKKs*, and 57 *MAPKKKs* that were expressed in *H. glycines-*infected roots of S54 (Fig. 5B; Table S13). Among these *MAPK* cascade genes, one *MAPKK* (*Glyma.15G172600*) and three *MAPKKK* (*Glyma.05G094400*, *Glyma.15G048500*, and *Glyma.17G245300*) genes were highly induced by *H. glycines* infection in S54, while expression of *Glyma.05G094400* was significantly down-regulated. The most strongly induced one of these four DEGs was *Glyma.15G048500*, whose expression was up-regulated by 10.7-fold in infected S54 roots and was unchanged in S67 up infection.

### Plant hormones involved in soybean defense signaling to *H. glycines* attack

In our study, the biosynthesis of jasmonic acid (JA), SA, ethylene (ET), and their mediated defense signaling were affected by *H. glycines* infection (Fig. 5B; Table S10). Briefly, the expression levels of several genes encoding key enzymes involved in the biosynthesis of JA, SA, and ET were significantly up-regulated (Fig. 5B), while gibberellic acid (GA) biosynthesis was suppressed. These enzymes included: 1) JA pathway: lipoxygenases (LOX*;* Glyma.07G006700, Glyma.08G189400, Glyma.10G153900, Glyma.13G030300), oxophytodienoate-reductase 3 (DDE1/OPR3) (Glyma.13G109800, and Glyma.17G049900)^29^, and JA methyltransferase genes (*Glyma.02G054200* and *Glyma.18G238800*), which are involved in converting JA to methyl jasmonate (MeJA)^30^; 2) ET pathway: two ethylene-forming enzymes (ACO4/EFE), one ACC oxidase (ACO) and ten 2-oxoglutarates (2OG) showing up-regulated expression, and ACC synthase 1 (ACS) and Fe (II)-dependent oxygenase (DMR6) showing varying expression responses to *H. glycines* infection; 3) SA pathway: EDS1, BON-associated proteins (BAPs), SA-binding proteins (SABPs), and cyclic nucleotide-gated ion channel (CNGC; Fig. 5B). However, the expression levels of several genes encoding GA oxidases (GAOX) and GA-induced protein (GASA) were suppressed (Fig. 5B).

In addition to the induction of phytohormone biosynthesis, genes involved in hormone-mediated defense signaling were significantly induced in S54 upon *H. glycines* infection (Fig. 5B; Tables S10). These DEGs included the essential genes participating in two major branches of the JA signaling pathway: the ERF branch and the MYC branch. We found that the ERF branch was induced while the MYC branch was suppressed upon *H. glycines* infection in S54. These genes included three pathogenesis-related (PR) proteins and several transcriptional regulators upstream of PR proteins in the ERF branch, such as three thioredoxin superfamily proteins (GRX480)^31^, and eight WRKY70s as PR-transcription activators^32^ (Fig. 5C; S12). In the MYC branch, seven DEGs encoding jasmonate-zim-domain protein 1 (JAZ1), a suppressor of the downstream transcriptional regulator MYC2^29^, were significantly induced by *H. glycines* infection in S54. Accordingly, ten of thirteen *MYC* DEGs were down-regulated (Fig. 5B; Table S10). Moreover, genes involved in SA signaling-mediated *H. glycines* resistance were also strongly induced in this study, such as *GmSAMT1* (*Glyma.02G054200*)^33^ and three NPR1-interacting proteins (NIMIN-1)^34^. Expression of *Glyma.02G054200* was up-regulated by 54.5-fold in infected S54 roots compared with the control, much higher than the 8.1-fold enhancement observed in S67 (Fig. 5B). Moreover, ET signaling genes, such as *ERF*, were also significantly induced (Fig. 5B).

### Transcription factors involved in transcriptional regulation during *H. glycines* infection

WRKY transcription factors have been identified as one the largest families of regulatory proteins, and there is increasing evidence indicating that WRKYs are involved in soybean defense responses to pathogens^35^ and soybean aphids^36^. In our study, we identified 83 *WRKY* genes that were expressed in S54 roots based on available annotations (Table S13), 33 (39.8%) of which were significantly induced after *H. glycines* infection (Fig. 5C; Table S11). We temporally classified these 33 *WRKY* genes into ten groups (*WRKY23*, *WRKY28*, *WRKY30*, *WRKY40*, *WRKY50*, *WRKY51*, *WRKY67*, *WRKY70*, *WRKY75*, and unassigned *WRKY* homologs) based on the annotated *WRKY* gene symbols in *Arabidopsis*. The groups *WRKY40* and *WRKY70* contained seven and eight DEGs, respectively, representing the top two largest *H. glycines*-induced *WRKY* groups. We found that the top five (*Glyma.13G370100*, *Glyma.14G102900*, *Glyma.14G103100*, *Glyma.17G22230*0, and *Glyma.17G222500*) strongly induced *WRKYs* in S54 (ranging from 9- to 32-fold changes) were *WRKY40* genes, which were involved in modulating the transcription regulation of stress-responsive nuclear genes^37, 38^. Notably, two *WRKY40* DEGs (*Glyma.14G103100* and *Glyma.17G222500*) with the most dramatic changes (32- and 23-fold) in expression in S54 showed only two-fold up-regulation in S67. In addition, expression of *Glyma.13G267600* was increased by six-fold after *H. glycines* infection in S54, representing the most strongly induced *WRKY70* (Fig. 5C). These results suggested that *WRKY* family genes might act positively to transcribe downstream target genes to establish resistance to HG type 2.5.7 infection. On the other hand, NAC and MYB, with demonstrated roles in plant defense responses to various environmental stresses^39–41^, were also strongly induced by HG type 2.5.7 infection (Fig. 5C; Table S11). After infection, 31 *MYB* genes and 18 *NAC* genes were significantly induced, the expression levels of 17 *MYB* genes and 13 *NAC* genes were significantly up-regulated in S54.

### Expression profiles of genes associated with cell wall integrity were strongly affected in S54 by HG type 2.5.7 infection

In our study, we found that the expression profiles of the majority of the genes that function in the synthesis of polysaccharides and cell wall integrity were significantly affected after *H. glycines* attack in S54 but not in S67 (Fig. 5D; Table S12). These DEGs included five cellulose synthase genes (*CESAs*) and 34 *FASCICLIN*-like arabinogalactans (*FLA*) (Table S12) that associated with cellulose deposition and subsequently affected cell wall architecture^42^, pectic enzymes involved in cell wall degradation^43^, and seven pectin methylesterase inhibitors (PMEIs) capable of decreasing PME activity^44^. In addition, the expression profiles of the genes involved in cell wall loosening were also affected by *H. glycines* infection in S67 (Fig. 5D; Table S12). These DEGs include six xyloglucan endotransglucosylase (*XET*) genes, which function in loosening the plant cell wall^45^, seven *EXP* or *EXPR* expansin genes^46^, and two pectin acetylesterase genes (*PAE*), which can modulate cell extensibility via PAE-mediated acetylation and deacetylation of pectin in cell wall^47^.

In contrast, genes related to cell wall strengthening and cell wall-related defense signaling were significantly induced in S54 but not in S67 (Fig. 5D). For example, two tandem-located *PGIP* genes (*Glyma.08G079100* and *Glyma.08G079200*) that are capable of inhibiting the pectin-depolymerizing activity of PGs^48^ were up-regulated, which was concomitant with a significant reduction in the expression of a *G. soja PG* gene (*Glyma.08G287400*). Regarding cell wall-associated defense signaling, the expression levels of six wall-associated kinase (*WAK)* genes^49^ were significantly induced by *H. glycines* infection in S54, with only one gene (*Glyma.14G158600*) showing strongly up-regulated expression in both S54 and S67 (Fig. 5D).

### Other defense-related proteins were induced by *H. glycines* infection

Chitinases have the capacity to digest chitin, an essential component of fungi and the exoskeletal elements of some animals, including worms and arthropods. In this study, the expression levels of eight chitinase genes were affected by *H. glycines* infection in S54, with six of them showing increased expression (Fig. 5D; Table S11). The degrees of up-regulation of four chitinase genes (*Glyma.02G042500*, *Glyma.02G007400*, *Glyma.11G124500*, and *Glyma.16G173000*) were much larger in infected S54 than those in S67. The *PR3*-like chitinase gene (*Glyma.02G042500*) was the most strongly induced: 26.9-fold up-regulation in S54 compared with a 2.9-fold change in S67 after infection. In addition, the *PR4* chitinase gene *Glyma.20G225200* was induced only in infected S54 roots. However, the expression levels of two chitinase-like protein (*CTL*) genes (*Glyma.09G038500* and *Glyma.15G143600*) that were related to lignin accumulation^50^ were decreased upon infection in S54.

The expression levels of genes encoding other classes of defense-related proteins were also induced by *H. glycines* infection in S54 (Fig. 5D; Table S12). These proteins include nine glutathione S-transferases (GSTs), a ubiquitous class of enzymes that are capable of detoxifying xenobiotics^51^; three heat shock proteins (HSPs), of which RTM2 protein (*Glyma.04G129900*) functions in phloem to redistrict long-distance movements of virus^52^, another *HSP* gene, *Glyma.04G108500*, that was strongly induced by 32.3-fold following HG type 2.5.7 infection in S54 but that was insensitive in S67; and eight protease inhibitors that have the capacity to interfere with the digestive processes of insects, leading to resistance reactions^53^. It was interesting to note that these eight protease inhibitor genes included three pairs of genes that were tandemly located on chromosomes 8, 10, and 20. Interestingly, the two major genes *rhg1* (*Glyma18g02580, Glyma18g02590, Glyma18g02580*) and *Rhg4* (*Glyma.08G108900*), conferring SCN resistance to HG type 0, didn’t show significant differences in expression between resistant (S54) and susceptible (S67) genotypes after SCN treatment (Fig. S7).

### Time-course gene expression pattern suggests the involvement of the DEGs in *H. glycines* resistance

To validate the results from our RNA-seq data, we selected 20 genes for *q*PCR assays in both S54 and S67 (Table S14). The fold changes of these genes obtained from *q*PCR assays were compared with RNA-seq results. A good correlation (*R* ^2^ = 0.94, *P* < 0.001, Fig. S6) between RNA-seq and *q*PCR results validated the accuracy and robustness of our RNA-seq results.

**Figure 6:**
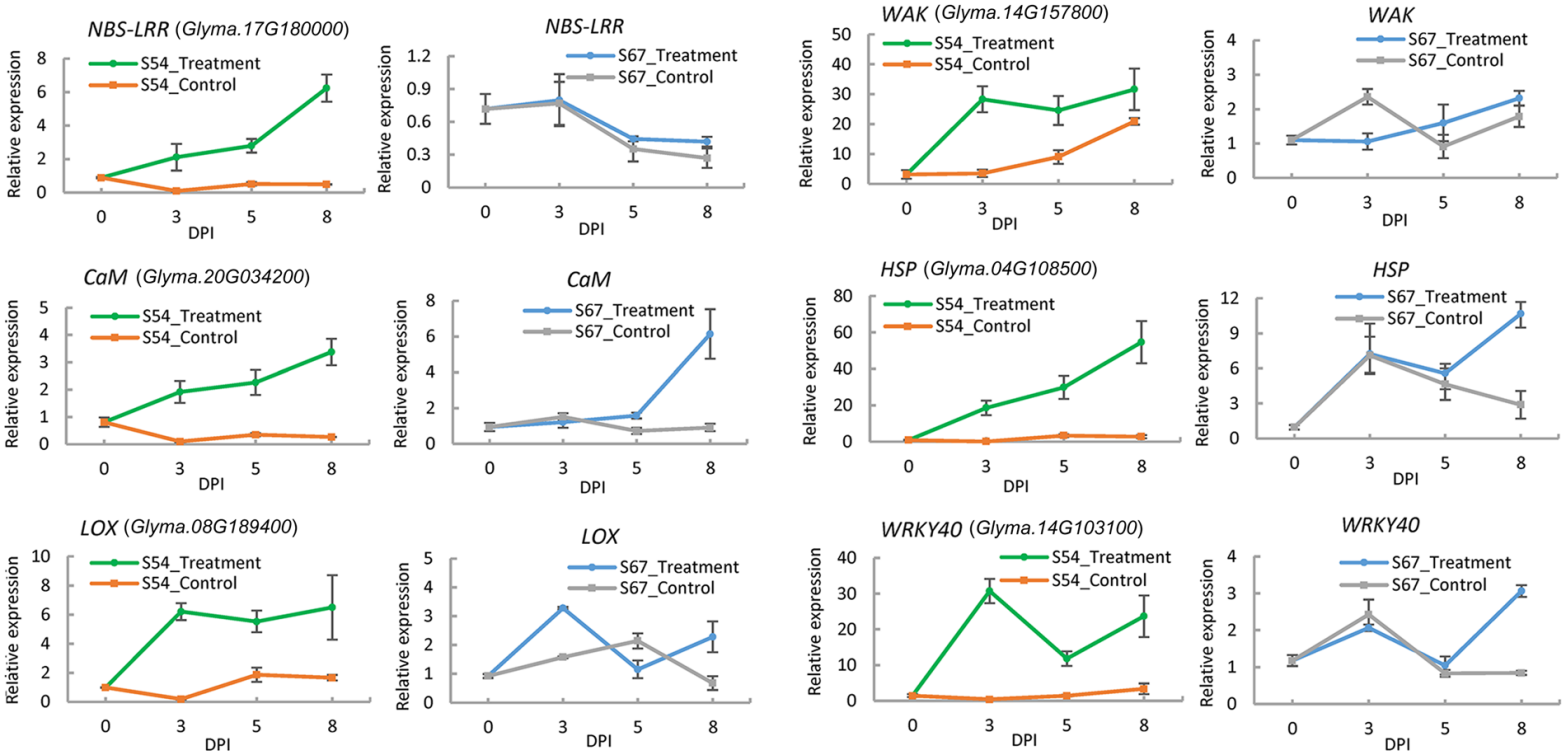
Dynamic expression levels of six representative genes at 0, 3, 5, and 8 dpi after *H. glycines* infection in S54 and S67. Error bars represent the standard deviation.

To obtain a better understanding of the expression patterns for DEGs during the interactions, six representative genes from different functional categories described above were selected, and *q*PCR was conducted in resistant S54 and susceptible S67 during the time course of 0, 3, 5, and 8 dpi (Fig. 6). These genes included *NBS-LRR* (*Glyma.17G180000*), involved in PTI; *CaM* (*Glyma.20G034200*), involved in calcium/calmodulin mediated defense signaling; *LOX* (*Glyma.08G189400*), involved in JA synthesis; *WAK* (*Glyma.14G157800*), involved in cell wall-related defense; *HSP* (*Glyma.04G108500*); and *WRKY40* (*Glyma.14G103100*), involved in transcription regulation. As shown in Fig. 6, the overall up-regulated expression patterns of these genes from *q*PCR during the testing time points were in good agreement with the RNA-seq results. The relative expression differences for all six genes in S54 were much larger than those in S67. As expected, the expression levels of all six genes, except gene *WRKY40*, were continuously increased in *H. glycines-*infected S54 roots from 3 to 8 dpi, with the highest induced levels in gene expression observed at 8 dpi. In contrast, these six genes showed insensitivity or reduced expression levels in S54 control roots, with an exception of *WAK* showing up-regulated expression at 5 and 8 dpi. It is important to note that marked up-regulation in the expression levels of *WAK*, *HSP*, and *WRKY40* was observed in *H. glycines-*infected S54 roots compared with either 0-d uninfected and the counterpart controls during the treatment. However, the expression patterns for these six genes in S67 were distinct as observed in S54. In S67, all six genes behaved similarly in *H. glycines-*infected S67 and control roots before 5 dpi, without showing significant fluctuations in expression following SCN infection. Significant inductions in expression profiles for the *CaM*, *HSP*, and *WRKY40* genes were observed in infected S67 roots at 8 dpi. These results further validated the accuracy and robustness of our RNA-seq results. The persistent up-regulation in expression for these genes in S54 suggested that these genes/alleles might positively be involved in *H. glycines* resistance. We might further speculate that genes belonging to these six functional categories or that are involved in related signaling pathways might also contribute to *H. glycines* resistance in S54, but this hypothesis needs further verification.

## Discussion

### The *G. soja* population represents an important exotic resource for enriching the gene pool for *H. glycines* management

It is known that crop wild relatives (CWRs) are exposed to wild environments that are harsher than the farm fields where cultivated crops usually grow. Long-term stress exposure and an evolutionary arms race between CWR and pests have mandated that CWRs possess a more sophisticated defense mechanism for adaption to various environments than their cultivated counterparts^54, 55^. Thus, CWRs hold great potential for providing exotic and novel genetic resources for crop biotic and abiotic stresses. In our study, the resistant *G. soja* S54 originated from East Asia where the *H. glycines* possibly originated^56^. Due to greater genetic diversity retained in the *G. soja* than in the *G. max* population^16^, more sophisticated or genotype-specific defense networks or genes resistant to *H. glycines* were expected to exist in S54. Our and other studies^18, 57, 58^ have indicated that the *G. soja* population might serve as a new and exotic genetic resource for developing soybean cultivars conferring *H. glycines* resistance. With in-depth transcriptomic analysis, we were able to identify the both previously identified genes (such as *GmSAMT1*) and a subset of novel genes (such as *WRKY40*, *NIMIN-1*, protease inhibitor-encoding genes *and* cell wall-related defense gene*s*) that might be involved in defense responses to *H. glycines* in *G. soja*. The compatible expression levels of the well-studied SCN-resistant genes *rhg1* and *Rhg4* between resistant (S54) and susceptible (S67) genotypes after SCN treatment suggested the interactions between plant and SCN can be complex and species/HG type specific. Our results also indicated the existence of a complex and coordinated signaling network occurring during the process of resistant *G. soja* defending against HG 2.5.7, further improving our comprehensive understanding of how the resistant *G. soja* copes with *H. glycines* attacks.

### Advantages of transcriptomic profiling strategies to measure soybean responses to *H. glycines* infection

It seems like inhibition of nematode development is a conserved resistance strategy, and 5 to 8 dpi appears to be a critical period to effectively initiate actions to defend against *H. glycines* in both *G. max* and *G. soja*. Instead of investigating single or several selected time points and syncytia^4, 6^, we examined the transcriptome changes in pooled samples of whole roots from 3, 5, and 8 dpi. The resulting FPKM values for each gene might actually reflect the average gene expression level for the three time points at a systematic level. Consistent results were also observed between the GO and KEGG analyses (Figs 4 and 5, S3; Table S6). Sequencing-based RNA-seq analysis holds additional advantages in determining the transcriptomic characteristics of soybean responses to *H. glycines* infection. Utilization of the uniquely mapped reads for measurement of gene expression enabled the comparison of expression levels of homologous genes accurately. As a result, our study produced a large amount of genome-wide gene expression data in *G. soja* after HG 2.5.7 infection and also provided novel expression patterns of homologous family genes and new genes that might not have been reported in previous related studies^3–7, 9, 15^.

### Both ETI and PTI are important in *G. soja* defense against *H. glycines* infection

**Figure 7:**
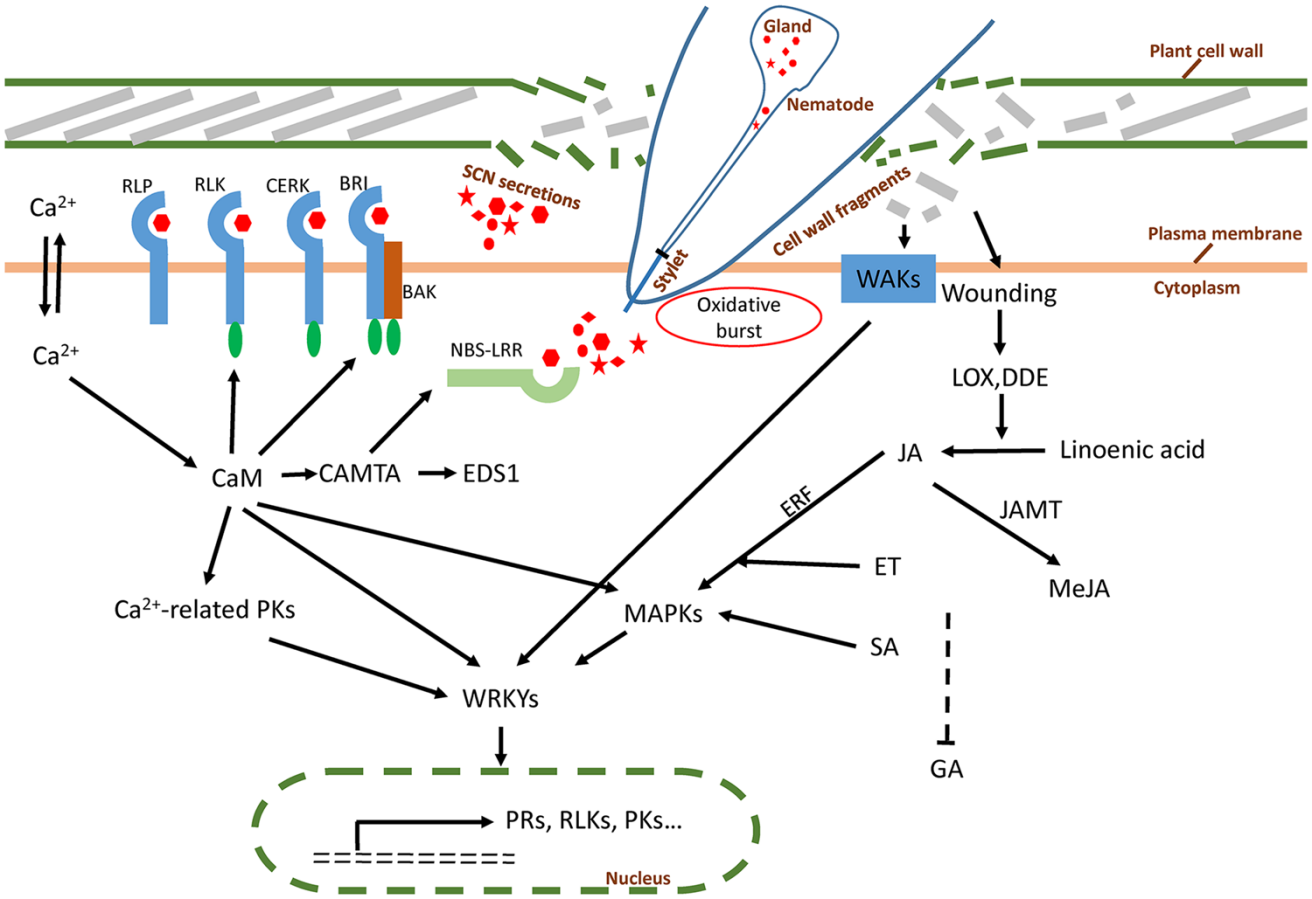
A proposed regulatory model to illustrate the defense response to HG type 2.5.7 infection in *Glycine soja*.

The current view of the plant immune system can be represented as a “zigzag” model, in which recognition of pathogen associated molecular patterns (PAMPs) or effectors, by host-encoded receptors^59^ triggers subsequent immune responses. Thus far, several nematode resistant genes (*Mi-1*, *Hero*, *Gpa2*, and *Gro1*) from other plant species have been identified as encoding NBS-LRR proteins^60^. However, the known genes (*rhg1* and *Rhg4*) that confer *H. glycines* resistance are not canonical NBS-LRR type genes^61^ in soybean. Identification of the *NBS-LRR* genes as DEGs in the present (Fig. 6A) and previous studies^3, 4, 7^ suggests that timely recognition of *H. glycines* secretions appears to be one of the important mechanisms soybeans employ to cope with *H. glycines*. As observed from continuous up-regulation of receptors (NBS-LRR and WAK) and other signaling genes in S54 as the infection period increased (Fig. 7), the recognition coupled with the rapid and effective induction of defense responses might be persistent throughout the interaction, which might make a difference between resistance and susceptibility.

### Calcium/calmodulin-mediated signaling might play a central role in coordinating various regulatory pathways in responses to *H. glycines* infection in *G. soja*

Ca^2+^/calmodulin has long been considered a crucial component in the wounding signaling pathway or mediating plant defense against various biotic attackers^62^. Cellular Ca^2+^ fluxes are among the earliest detectable biochemical features upon pathogen or microbe recognition^63^. However, it was surprising that Ca^2+^ sensors have been only sporadically reported in *H. glycines-*soybean interactions^3, 4, 7^. Significant induction of a number of Ca^2+^-binding proteins (calmodulin; Fig. 6A) and persistent increases in the expression of *CaM* (*Glyma.20G034200*; Fig. 7) suggests an increasing cellular Ca^2+^ concentration upon *H. glycines* infection in roots, and this concentration might achieve the threshold to bind or trigger the functions of calmodulin^63^.

Calcium/calmodulin signaling has been closely linked to PTI- and ETI- associated *H. glycines* resistance. For example, Ca^2+^-dependent protein kinases can phosphorylate WRKY8, WRKY28 and WRKY48, leading to direct post-translational regulation of the TF activities^64^. Furthermore, it has been demonstrated that the different Ca^2+^ amplitudes might be involved in the coordination of several signaling branches during or after the detection of PAMPs, such as flg22, elf18, and chitin, during defense response^65^. Consistently, strong inductions of various types of PKs, receptors (Fig. 5A), chitinases (Fig. 5D), and calmodulins upon *H. glycines* infection suggest that these phosphorylation-mediated signaling pathways might be coordinated by different Ca^2+^ amplitudes or might temporally stimulate other Ca^2+^-dependent signal flows to efficiently respond to the infection. In addition, CAMTA3, the best characterized calmodulin-regulated transcription factor, can transcriptionally regulate EDS1 and TIR-NBS-LRR mediated defenses in *Arabidopsis* ^66^. Thus, identification of various types of receptors and calmodulin simultaneously in this study suggests a tight link between calcium/calmodulin signaling with PTI and ETI to *H. glycines* infection. We speculate that Ca^2+^/calmodulin-mediated regulation might function as a dispatcher in orchestrating a complex interplay between these regulatory pathways to establish a resistance response to *H. glycines* infection.

### The endogenous plant signaling molecules SA, JA and ET are coordinately involved in soybean defense against *H. glycines* infection

Plant hormones (SA, JA, and ET) play important roles in modulating plant defenses against various diseases and pests^67^. Although both different and consistent expression patterns of JA/ET biosynthesis genes were found compared to previous studies^4, 68^, our and previous studies^3, 4, 6–9, 15^ suggested that SA, JA and ET signaling genes might be the conserved defense mechanisms involved in *H. glycines* resistance in both *G. max* and *G. soja*. A dramatic increase in the expression of SA, JAs and ET signaling genes might contribute to *H. glycines* resistance, coinciding with a recent study showing that these hormones were involved in systemic defenses in rice against the root knot nematode (*Meloidogyne graminicola*)^68^. In addition, involvement of SA signaling in SCN resistance was shown by a recent study in which overexpression of the *SAMT* in soybean conferred *H. glycines* resistance in both lab^33^ and field tests^69^.

On the other hand, in contrast to the finding that SA and JA/ET signaling are mutually antagonistic^70^, JA/ET and SA signaling might function synergistically in defending against *H. glycines* infection in S54, as observed previously^70, 71^. The positive and negative cross-talk between SA and JA/ET signaling may be regulated by the nature of the pathogen^67^. The cross talk between these signaling pathways was also indicated by the identification of the genes involved in both SA and JA pathways in this study, such as *GRX480* ^31^ and *PR1* ^72^, providing optimal hormone-mediated defense.

### Quantitative differences in gene expression might cause the differences between resistant and susceptible responses to *H. glycines*

Our results suggest that the establishment of the resistance responses is fulfilled by massive changes in the expression profiles of over 2,000 genes occurring at the whole-genome level. Similar shapes but different amplitudes of the expression profiles for these genes between S54 and S67 (Fig. 4) might result in major differences between resistant and susceptible responses to *H. glycines*. Whether the changes were caused by one major locus or many loci with small effects could be addressed by further studies using linkage mapping. Furthermore, this quantitative resistance to *H. glycines* might also be reflected by more active energy metabolism in S54 than S67, since turning on effective defense is energy intensive, and a decrease in the energy reserve might result in insufficient energy for the full expression of defense mechanisms^73^.

Soybean responses to *H. glycines* are complex, and a number of genes function differentially in this dynamic interaction. Although we presented several signaling and defense-related pathways by simply categorizing the genes based on the current knowledge, the regulatory network for *H. glycines* resistance is far more complex. Each signaling pathway presented here is not independent of the others because several important convergence points were identified here. For example, WRKY70, GRX480, and MAPK are involved in SA and JA cross talk^32, 67, 74^, and MAPKs are also regulated by Ca^2+^/calmodulin^62^. The feed-back and feed-forward loops generated by these nodes result in increased signaling complexity but provide effective plasticity in defending against *H. glycines*. Collectively, the signaling mechanism associated with *H. glycines* resistance is likely to be a network of highly interconnected pathways by which resistant *G. soja* are able to effectively fend off *H. glycines* in a plastic manner.

### Hypothesized model of the SCN resistance network in *G. soja*

Based on our results, we propose a model to summarize the resistance-associated defense response in wild soybean (Fig. 7). Before establishing a permanent feeding site close to the vasculature, an *H. glycines* J2 has to penetrate the root epidemic cells by dismantling the cell walls. During cell probing and syncytia development, the *H. glycines* J2 releases various types of proteins to facilitate nematode migration and syncytia development^75^. As one of the earliest responses to pathogen attack, oxidative burst-related defense responses might occur in both resistant and susceptible reactions to *H. glycines* infection^76^. In the resistant response, the cells in the *H. glycines*-penetrating path release wounding signals (i.e., cell wall fragments) upon penetration, and the breakdown of cell wall parts, such as pectin fragments, could be perceived by cell wall-related receptors in the resistant host, such as WAKs^49^. Wounding caused by nematode probing induces JA and SA synthesis and synergistic expression of JAs/ET and SA signaling, which has been shown to be an essential defense mechanism in plants coping with insect and pathogen attacks^29^. Expression of the GA-related pathway was coordinately suppressed. Meanwhile, the probing and giant cell-developing activities or released virulence proteins also induce a series of calcium-related signaling cascades. The resistant plant deploys Ca^2+^/calmodulin-mediated signaling to coordinate large cohorts of genes involved in protein phosphorylation (such as various types of PKs), transcriptional regulation (such as CAMTA and WRKYs) and ETI-associated defenses (such as NBS-LRRs) to provide the “best” defense response to *H. glycines* infection.

## Materials and Methods

### Plant materials

Two *G. soja* genotypes, PI 424093 (designated as S54 in this study) and PI 468396B (designated as S67 in this study), from the USDA Soybean Germplasm Collection were used in all experiments. S54 was identified as highly resistant (Female Index = 5.2%), and S67 showed high susceptibility (Female Index = 149%) to HG type 2.5.7 in our recent study^18^.

### Preparation of materials and SCN inoculation

Seeds of each genotype were surface sterilized in 0.5% sodium hypochlorite for 1 min and then rinsed and germinated on pieces of sterile filter paper with appropriate levels of sterile water in petri dishes for 3–4 days. Each healthy seedling was transplanted into a cone-tainer (Greenhouse Megastore, Danville, IL, USA) filled with sterile sand. All seeding-containing cones were pre-arranged in a cone-tainer tray (Greenhouse Megastore, Danville, IL, USA) using a randomized complete block design. All plants were maintained in the growth chamber (Percival, Perry, IA, USA) at 27 °C with 50% relative humidity and a long-period day light cycle of 16 h light/8 h dark. Seedlings were regularly watered daily to maintain plant moisture.

The HG Type 2.5.7 nematodes were reared on soybean cv. Hutcheson in the greenhouse under controlled temperature conditions (27 °C) and photoperiod (16 h light/8 h dark) for more than 30 generations. Female nematodes were harvested from stock roots by massaging the roots in water and sieving the solution through nested 850- and 250-µm sieves. The collected females were crushed with a rubber stopper in an 8-inch diameter 250-µm sieve, and the released eggs were collected in a 25-µm mesh sieve. The eggs were purified further by sucrose flotation^77^ with some modifications. The purified eggs were placed on wet paper tissues and incubated in a plastic tray with 1 cm of water. The tray was covered with aluminum foil and incubated at 27 °C. Three days after hatching, the second-stage juvenile nematodes (J2) were collected and concentrated into a final concentration of 1,800 J2/ml in a 0.09% agarose suspension. Three days after transplantation, the healthy and uniform seedlings were inoculated with 1 ml of J2 inoculum. In parallel, seedlings inoculated with 0.09% agarose were used as the controls.

To capture transcript variation responses to HG type 2.5.7 in *G. soja*, we pooled equal amounts of root samples for infected and control plants, respectively, at 3, 5, and 8 dpi for transcriptome quantification. Briefly, roots were excised from both inoculated and non-inoculated controls at 0, 3, 5, and 8 dpi, washed, flash frozen in liquid nitrogen, and stored at −80 °C until use. Four uniform individuals were pooled as one biological replicate, and at least four replicates were collected for each genotype at each time point. To confirm the successful infection, at least three root individuals at each time point were randomly selected and acid fuchsin-stained^78^ to observe the SCN growth at different stages.

### Library construction and Illumina RNA-seq

An equal amount of root tissues from each of three replicates per time-point (3, 5, and 8 d) was pooled for RNA extraction. Total RNA was extracted using an RNeasy mini total RNA isolation kit (Qiagen, Valencia, CA, USA), and RNA integrity, purity, and concentrations were assessed using an Agilent 2100 Bioanalyzer with an RNA 6000 Nano Chip (Agilent Technologies, Palo Alto, CA, USA). Purification of messenger RNA (mRNA) was performed using the oligo-dT beads provided in the NEBNext Poly(A) mRNA Magnetic Isolation Module (New England BioLabs, Beverly, MA, USA). Complementary DNA (cDNA) libraries for Illumina sequencing were constructed using the NEBNext Ultra Directional RNA Library Prep Kit (NEB, Beverly, MA, USA) and NEBNext Multiplex Oligos for Illumina (NEB, Beverly, MA, USA) using the manufacturer-specified protocol. Briefly, the mRNA was chemically fragmented and primed with random oligos for first-strand cDNA synthesis. The double-stranded cDNA was then purified, end repaired and “a-tailed” for adapter ligation. Following ligation, the samples were selected and sample-specific indexed. The final quantified libraries were pooled in equimolar amounts for sequencing on an Illumina HiSeq. 2500 utilizing a 125-bp read length with v4 sequencing chemistry (Illumina, San Diego, CA, USA).

### Sequence alignment and differential expression analysis

Quality control of reads was accessed by running the FastQC program (version 0.11.5), and Trimmomatic (version 0.36)^79^. High-quality reads were mapped against the Williams 82 soybean reference genome *Glycine max* Wm82.a2.v1^80^ with TopHat (version 2.1.1)^81^ using the minimum intron size (-i) parameter 30 and the maximum intron size (-I) 15000 as previously described^82^. Cufflinks (version 2.2.1)^83^ was used to estimate the gene expression (fragments per kilobase of transcript per million mapped reads – FPKM) levels. Only those genes with more than a 2-fold change and with a false discovery rate (FDR) ≤ 0.01 were considered significant differentially expressed genes (DEGs).

### Gene ontology (GO) and Kyoto Encyclopedia of Genes and Genomes (KEGG) pathway analysis

A heatmap was generated in R Bioconductor using the heatmap.2 function of the gplots package (https://cran.r-project.org/web/packages/gplots/). All of the DEGs determined using the above criteria were loaded into GO enrichment web tool at SoyBase (http://www.soybase.org) to identify enriched GO terms related to soybean responses to SCN HG type 2.5.7, as previously described^84^. KEGG pathway^85–87^ enrichment of DEGs was performed on the KEGG Orthology Based Annotation System (KOBAS version 2.0)^88^. Both GO terms and KEGG pathways with *q* ≤ 0.05 were considered significant enrichments. A Venn diagram was generated using the web tool Venny^89^. Assignment of the defense-related genes to the corresponding protein families was performed according to the gene annotation in the reference genome of Williams 82 (Wm82.a2.v1), and manually verified using online tools in SMART (http://smart.embl-heidelberg.de/) and Pfam (http://pfam.xfam.org/).

### RNA extraction and real-time *q*PCR analysis

For validation of RNA-seq results, we used the biological replicates of RNAs that were used for transcriptome sequencing to conduct *q*PCR. RNA was extracted using an RNeasy Plant mini Kit (Qiagen, Hilden, Germany) and quantified using a NanoDrop 2000 (Thermo Fisher Scientific, Wilmington, DE, USA). One microgram of RNA was treated with DNase I (Thermo Fisher Scientific, Wilmington, DE, USA) to remove any contaminated DNA before performing reverse transcription. Reverse transcription reactions were conducted using a RevertAid First Strand cDNA Synthesis Kit (Thermo Fisher Scientific, Wilmington, DE, USA) according to the manufacturer’s instructions.

A total of 20 genes were randomly selected for *q*PCR validation of the RNA-seq results. The soybean Ubiquitin 3 gene (GmUBI-3, Accession D28123) was used as an endogenous control. Intron-spanning primers were used to check for genomic DNA contamination. All gene-specific primers were designed for *q*PCR using the Primer3 web tool (version 0.4.0, http://bioinfo.ut.ee/primer3-0.4.0/). All primers are listed in supplemental Table S14. *q*PCR was performed on an ABI 7500 Fast real-time PCR system (Applied Biosystems, Foster City, CA, USA) using PerfeCTa^TM^ SYBR® Green FastMix^TM^ (Quanta Biosciences, Gaithersberg, MD, USA). Three biological replicates for each sample were used for *q*PCR analysis, and three technical replicates were analyzed for each biological replicate. The ΔΔCT method was used for relative quantification of gene expression^90^.

## Electronic supplementary material

Supplemental_Figures

Supplementary Dataset 1

Supplementary Dataset 2

Supplementary Dataset 3

Supplementary Dataset 4

Supplementary Dataset 5

Supplementary Dataset 6

Supplementary Dataset 7

Supplementary Dataset 8

Supplementary Dataset 9

Supplementary Dataset 10

Supplementary Dataset 11

Supplementary Dataset 12

Supplementary Dataset 13

Supplementary Dataset 14

Supplementary Dataset 15

Supplementary Dataset 16
