# Supplementary material for: Comparative RNA-Seq Analysis Uncovers a Complex Regulatory Network for Soybean Cyst Nematode Resistance in Wild Soybean (Glycine soja)
Source: Sci Rep. 2017 Aug 29;7:9699. doi: 10.1038/s41598-017-09945-0 (PMC5575055; doi:10.1038/s41598-017-09945-0)
Supplement: Supplementary file 1 — Supplemental_Figures [file 41598_2017_9945_MOESM1_ESM.pdf]

Comparative RNA-Seq Analysis Uncovers a Complex Regulatory Network for Soybean Cyst Nematode Resistance in Wild Soybean (*Glycine soja*)

Hengyou Zhang[1], Susanne Kjemtrup-Lovelace[2], Changbao-Li[3], Yan Luo[1, 4,] , Lars Chen[5], and Bao-Hua Song[1]*

[1]Department of Biological Sciences, University of North Carolina at Charlotte, Charlotte, NC, 28223, USA
[2]Department of Plant and Microbial Biology, North Carolina State University, Raleigh, NC, 27709, USA
[3]Double Haploid Optimization Group, Monsanto Company, St. Louis, MO 63167 USA
[4] Xishuangbanna Tropical Botanical Garden, Chinese Academy of Sciences, Yunnan, 650221 China
[5] Biology Department, University of North Carolina, Chapel Hill. NC 27599, USA

*Corresponding authors: bsong5@uncc.edu

SUPPLEMENTAL MATERIALS

Legends of supplemental figures

Figure S1 Volcano plots of differentially expressed genes in infected S54 and S67 compared with controls.

Figure S2 Dendrograms were constructed using differentially expressed genes. Treated and control samples were clustered in their respective groups.

Figure S3 Comparison of GO term enrichment for DEGs in S54 and S67. (A) Comparison of GO term enrichment for up-regulated DEGs between S54 and S67. (B) Comparison of GO term enrichment for down-regulated DEGs between S54 and S67.

Figure S4 Cell component analysis for 1,307 up- (A) and 1,304 down-regulated (B) DEGs.

Figure S5 Illustration of DEGs assigned in two over-representative KEGG pathways. (A) Phenylpropanoid biosynthesis. (B). Plant-pathogen interaction pathway.

Figure S6 Correlation analysis of fold changes for twenty genes between qPCR and RNA-seq results. Firgure

Figure S7 Comparison in expression of *rhg1* and *Rhg4* in S54 and S67 at 5 dpi. *rhg1-2580, Glyma18g02580/Glyma.18G022400; rhg1-2590, Glyma18g02590/Glyma.18G022500; rhg1-2610, Glyma18g02580/Glyma.18G022700; Rhg4, Glyma08g11490/Glyma.08G108900*

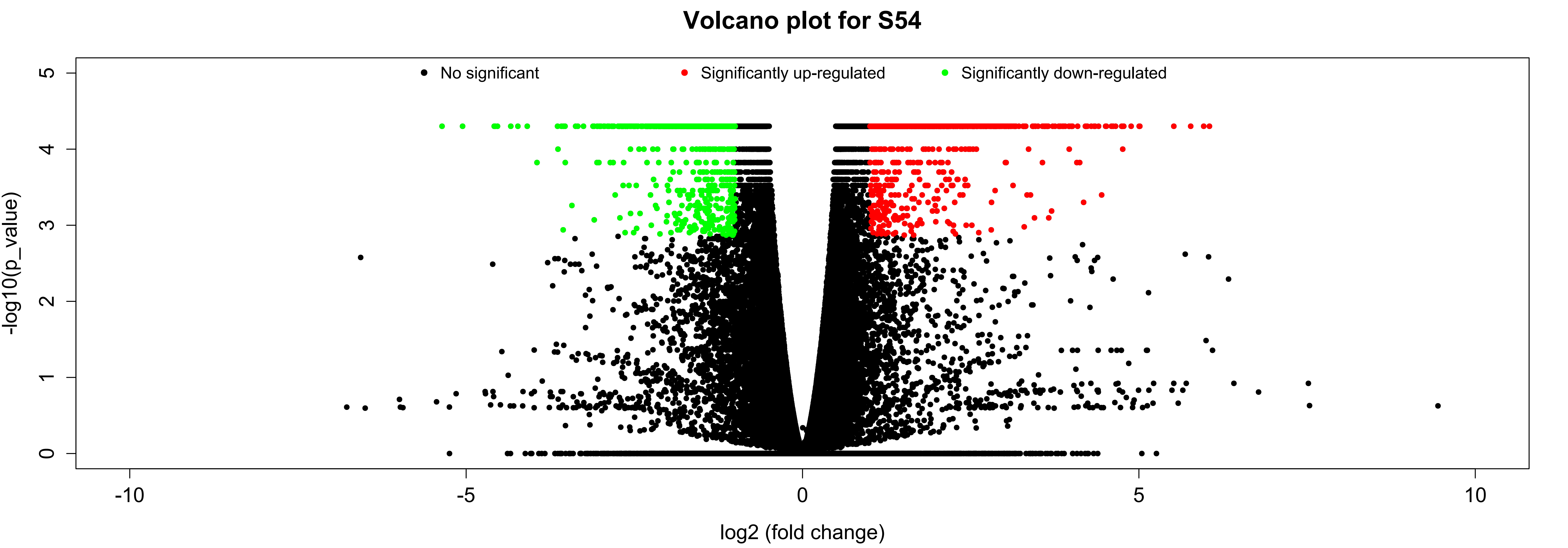

Volcano plot for S54
No significant
Significantly up-regulated
Significantly down-regulated
-log10(p_value)
0
1
2
3
4
5
-10
-5
0
5
10
log2 (fold change)

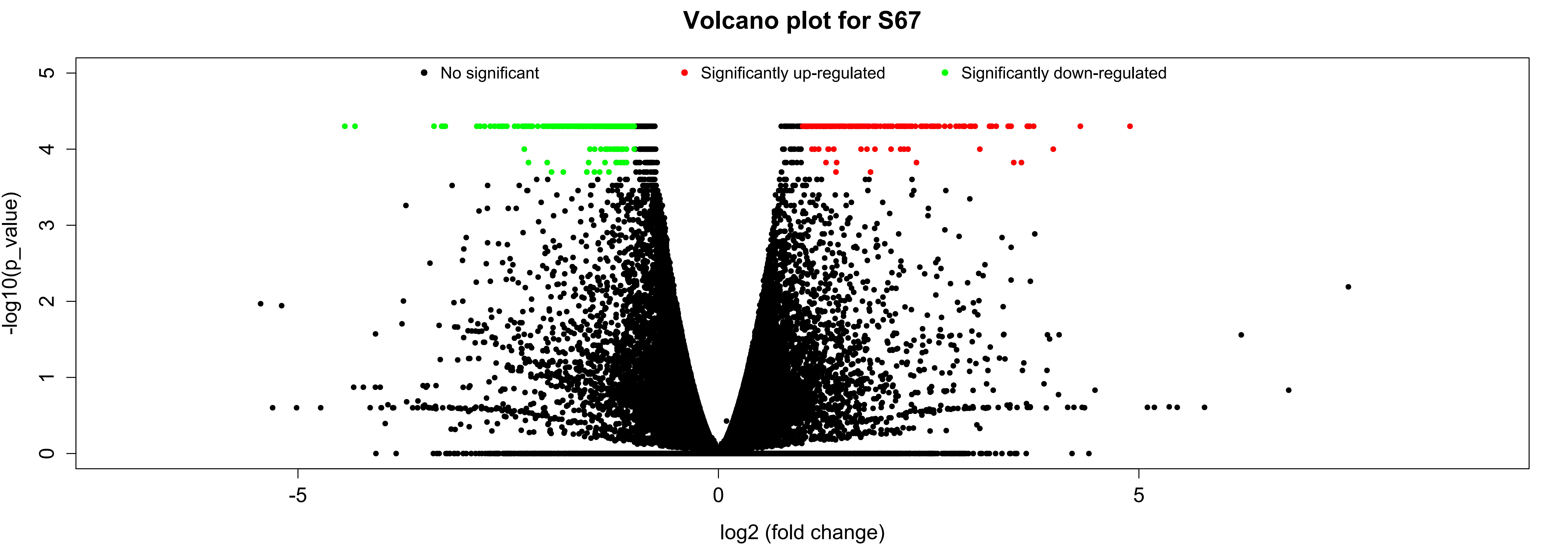

Volcano plot for S67
No significant
Significantly up-regulated
Significantly down-regulated
-log10(p_value)
0
1
2
3
4
5
-5
0
5
log2 (fold change)

a

b

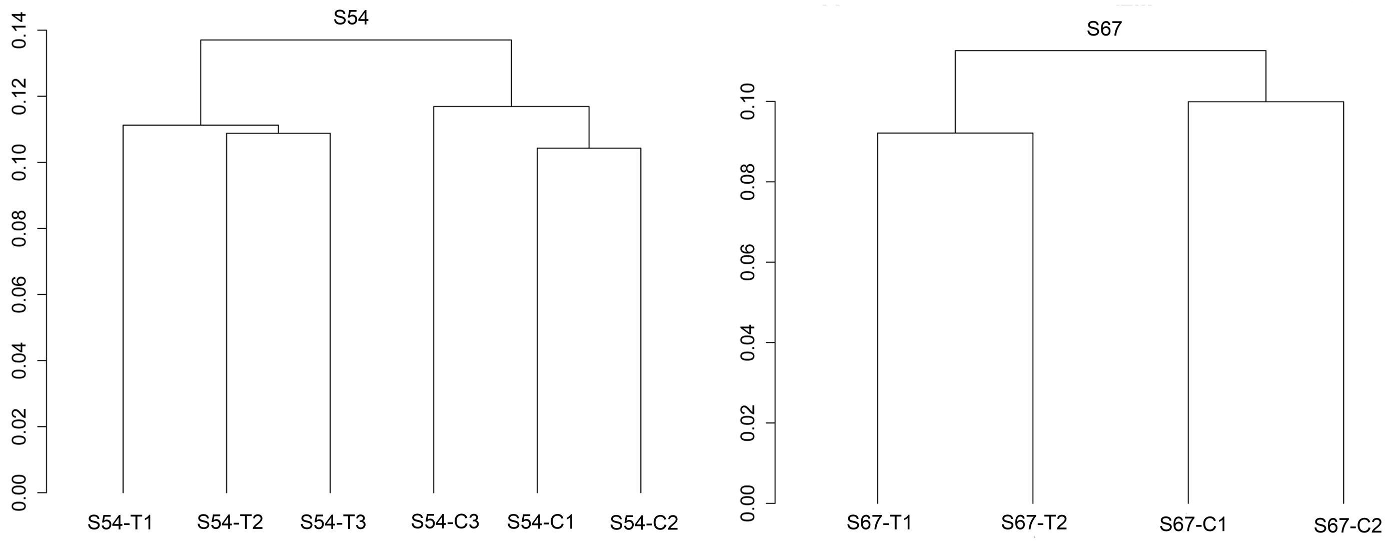
S54
0.00
0.02
0.04
0.06
0.08
0.10
0.12
0.14
S54-T1
S54-T2
S54-T3
S54-C3
S54-C1
S54-C2
S67
0.00
0.02
0.04
0.06
0.08
0.10
S67-T1
S67-T2
S67-C1
S67-C2

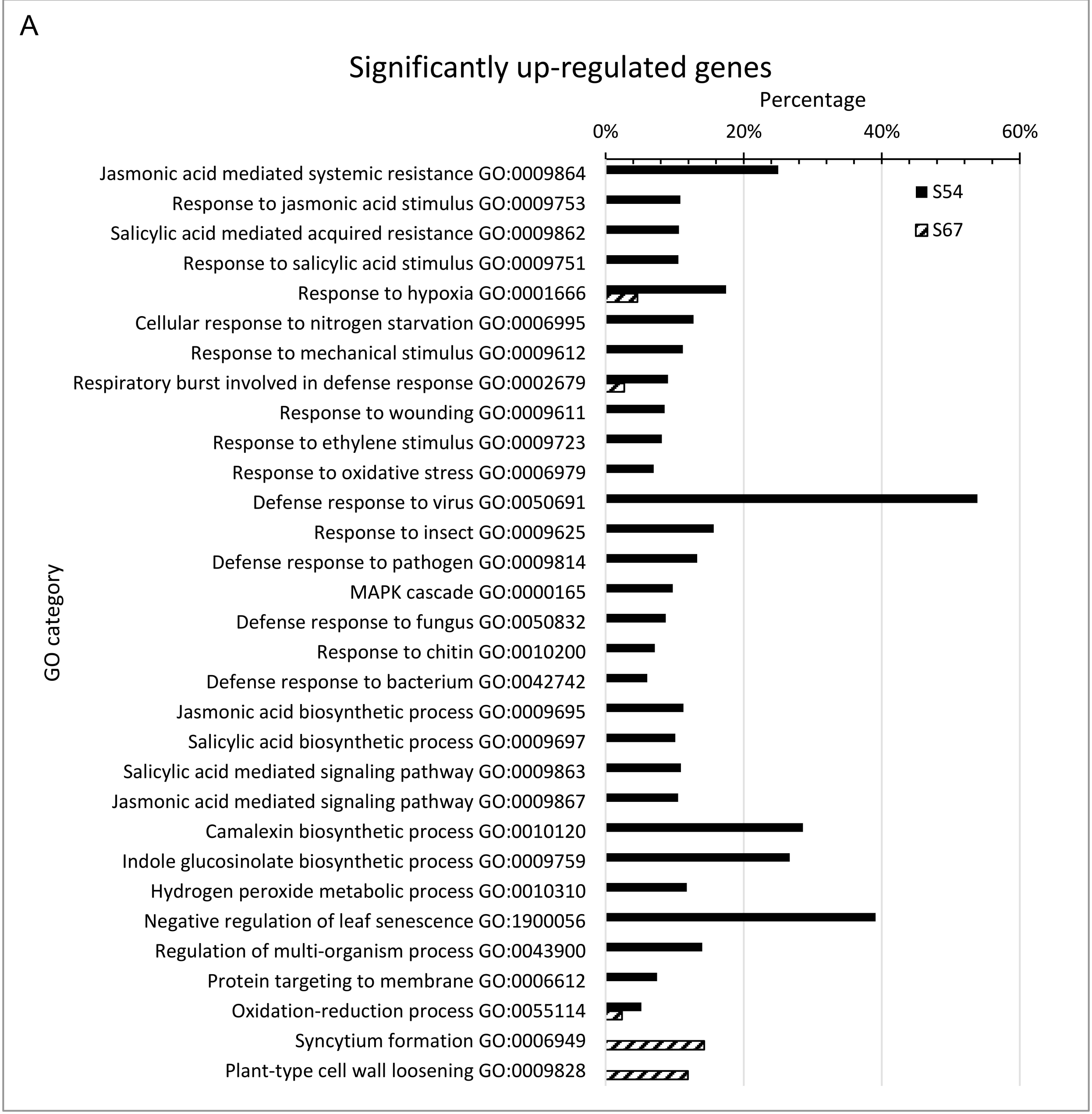
A
Significantly up-regulated genes
Percentage
0%
20%
40%
60%
S54
S67
GO category
Jasmonic acid mediated systemic resistance GO:0009864
Response to jasmonic acid stimulus GO:0009753
Salicylic acid mediated acquired resistance GO:0009862
Response to salicylic acid stimulus GO:0009751
Response to hypoxia GO:0001666
Cellular response to nitrogen starvation GO:0006995
Response to mechanical stimulus GO:0009612
Respiratory burst involved in defense response GO:0002679
Response to wounding GO:0009611
Response to ethylene stimulus GO:0009723
Response to oxidative stress GO:0006979
Defense response to virus GO:0050691
Response to insect GO:0009625
Defense response to pathogen GO:0009814
MAPK cascade GO:0000165
Defense response to fungus GO:0050832
Response to chitin GO:0010200
Defense response to bacterium GO:0042742
Jasmonic acid biosynthetic process GO:0009695
Salicylic acid biosynthetic process GO:0009697
Salicylic acid mediated signaling pathway GO:0009863
Jasmonic acid mediated signaling pathway GO:0009867
Camalexin biosynthetic process GO:0010120
Indole glucosinolate biosynthetic process GO:0009759
Hydrogen peroxide metabolic process GO:0010310
Negative regulation of leaf senescence GO:1900056
Regulation of multi-organism process GO:0043900
Protein targeting to membrane GO:0006612
Oxidation-reduction process GO:0055114
Syncytium formation GO:0006949
Plant-type cell wall loosening GO:0009828

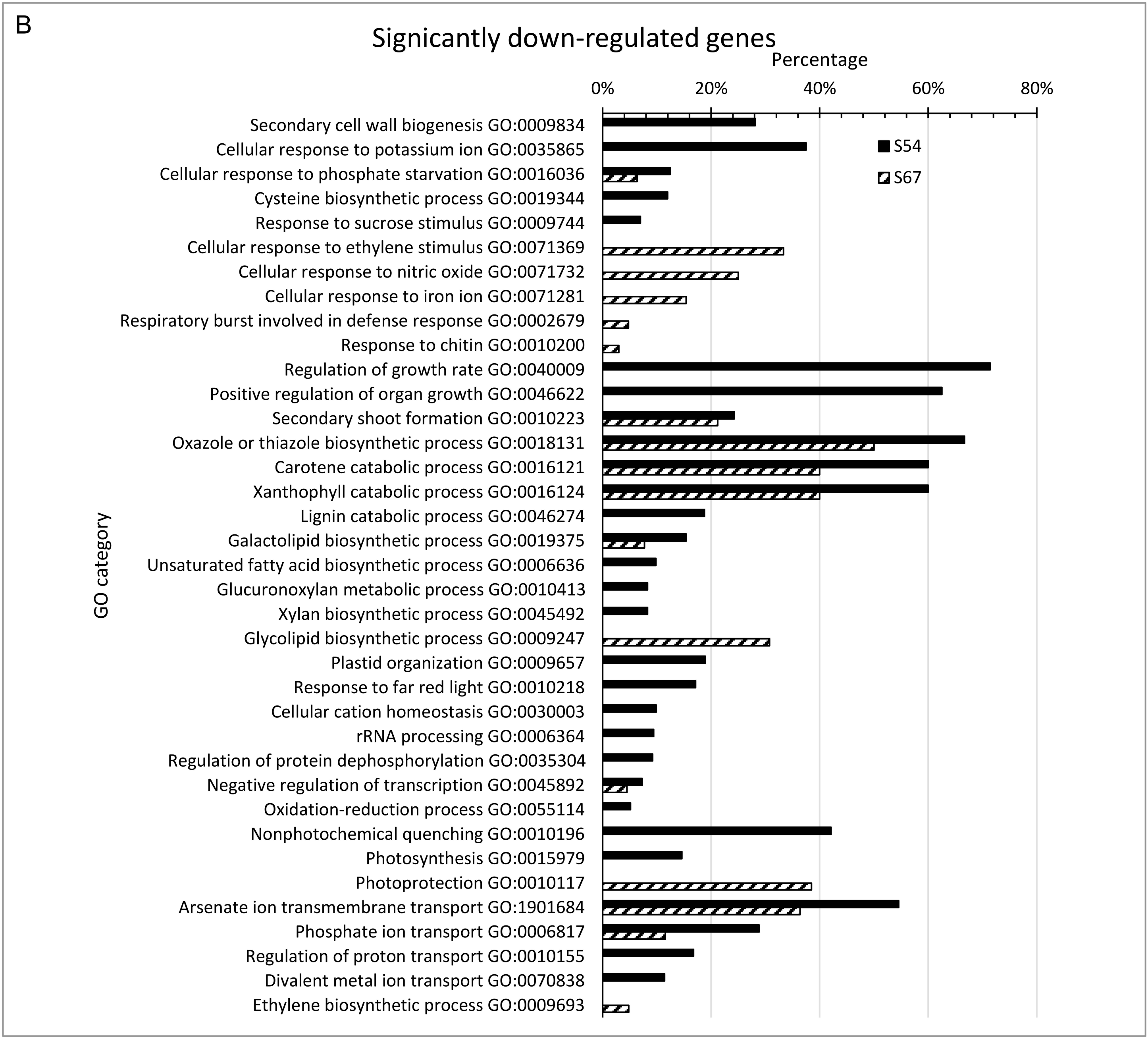
B
Signicantly down-regulated genes
Percentage
0%
20%
40%
60%
80%
S54
S67
GO category
Secondary cell wall biogenesis GO:0009834
Cellular response to potassium ion GO:0035865
Cellular response to phosphate starvation GO:0016036
Cysteine biosynthetic process GO:0019344
Response to sucrose stimulus GO:0009744
Cellular response to ethylene stimulus GO:0071369
Cellular response to nitric oxide GO:0071732
Cellular response to iron ion GO:0071281
Respiratory burst involved in defense response GO:0002679
Response to chitin GO:0010200
Regulation of growth rate GO:0040009
Positive regulation of organ growth GO:0046622
Secondary shoot formation GO:0010223
Oxazole or thiazole biosynthetic process GO:0018131
Carotene catabolic process GO:0016121
Xanthophyll catabolic process GO:0016124
Lignin catabolic process GO:0046274
Galactolipid biosynthetic process GO:0019375
Unsaturated fatty acid biosynthetic process GO:0006636
Glucuronoxylan metabolic process GO:0010413
Xylan biosynthetic process GO:0045492
Glycolipid biosynthetic process GO:0009247
Plastid organization GO:0009657
Response to far red light GO:0010218
Cellular cation homeostasis GO:0030003
rRNA processing GO:0006364
Regulation of protein dephosphorylation GO:0035304
Negative regulation of transcription GO:0045892
Oxidation-reduction process GO:0055114
Nonphotochemical quenching GO:0010196
Photosynthesis GO:0015979
Photoprotection GO:0010117
Arsenate ion transmembrane transport GO:1901684
Phosphate ion transport GO:0006817
Regulation of proton transport GO:0010155
Divalent metal ion transport GO:0070838
Ethylene biosynthetic process GO:0009693

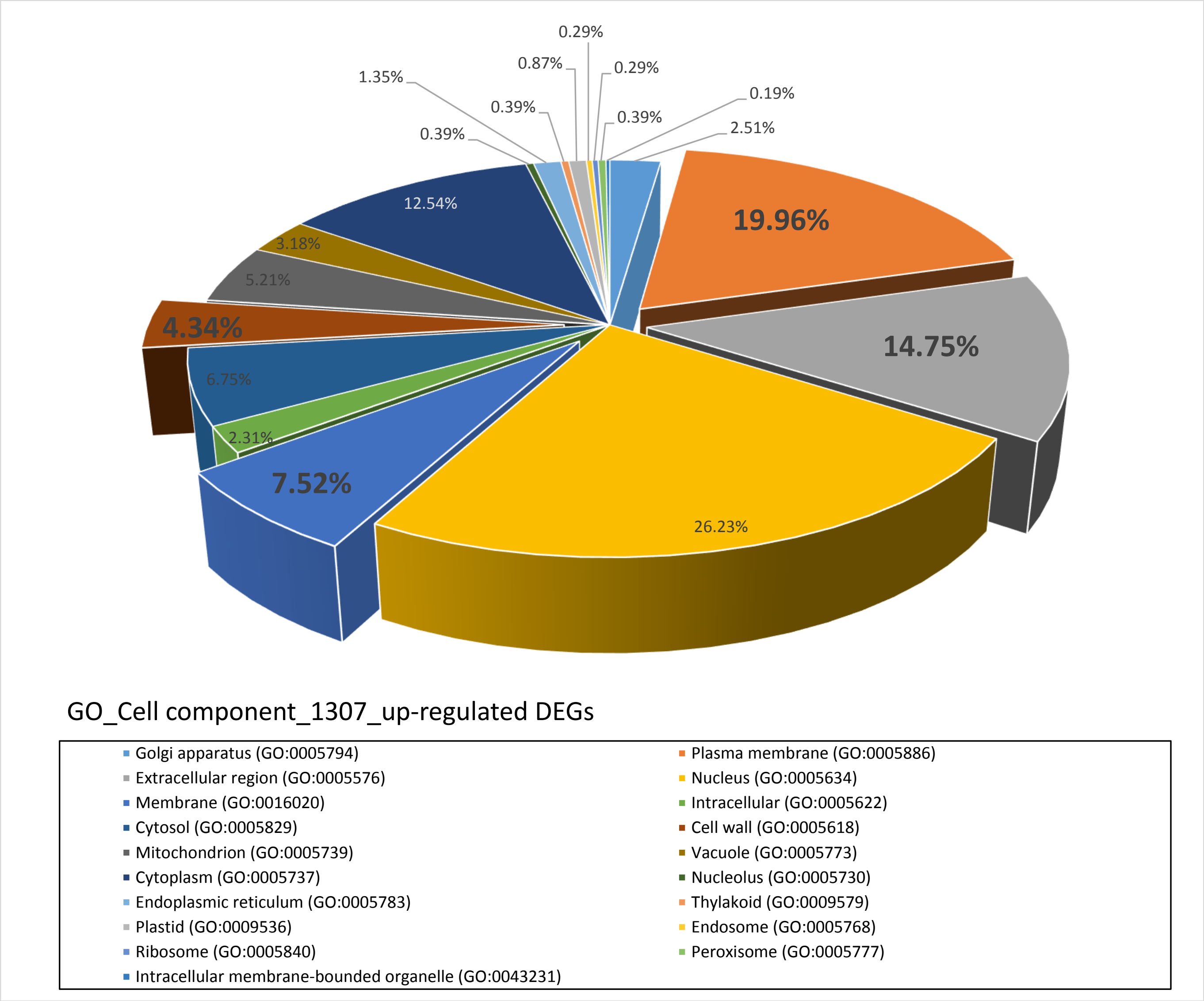
0.29%
0.87%
0.29%
1.35%
0.19%
0.39%
0.39%
2.51%
0.39%
19.96%
12.54%
3.18%
5.21%
4.34%
14.75%
6.75%
2.31%
7.52%
26.23%
GO_Cell component_1307_up-regulated DEGs
Golgi apparatus (GO:0005794)
Plasma membrane (GO:0005886)
Extracellular region (GO:0005576)
Nucleus (GO:0005634)
Membrane (GO:0016020)
Intracellular (GO:0005622)
Cytosol (GO:0005829)
Cell wall (GO:0005618)
Mitochondrion (GO:0005739)
Vacuole (GO:0005773)
Cytoplasm (GO:0005737)
Nucleolus (GO:0005730)
Endoplasmic reticulum (GO:0005783)
Thylakoid (GO:0009579)
Plastid (GO:0009536)
Endosome (GO:0005768)
Ribosome (GO:0005840)
Peroxisome (GO:0005777)
Intracellular membrane-bounded organelle (GO:0043231)

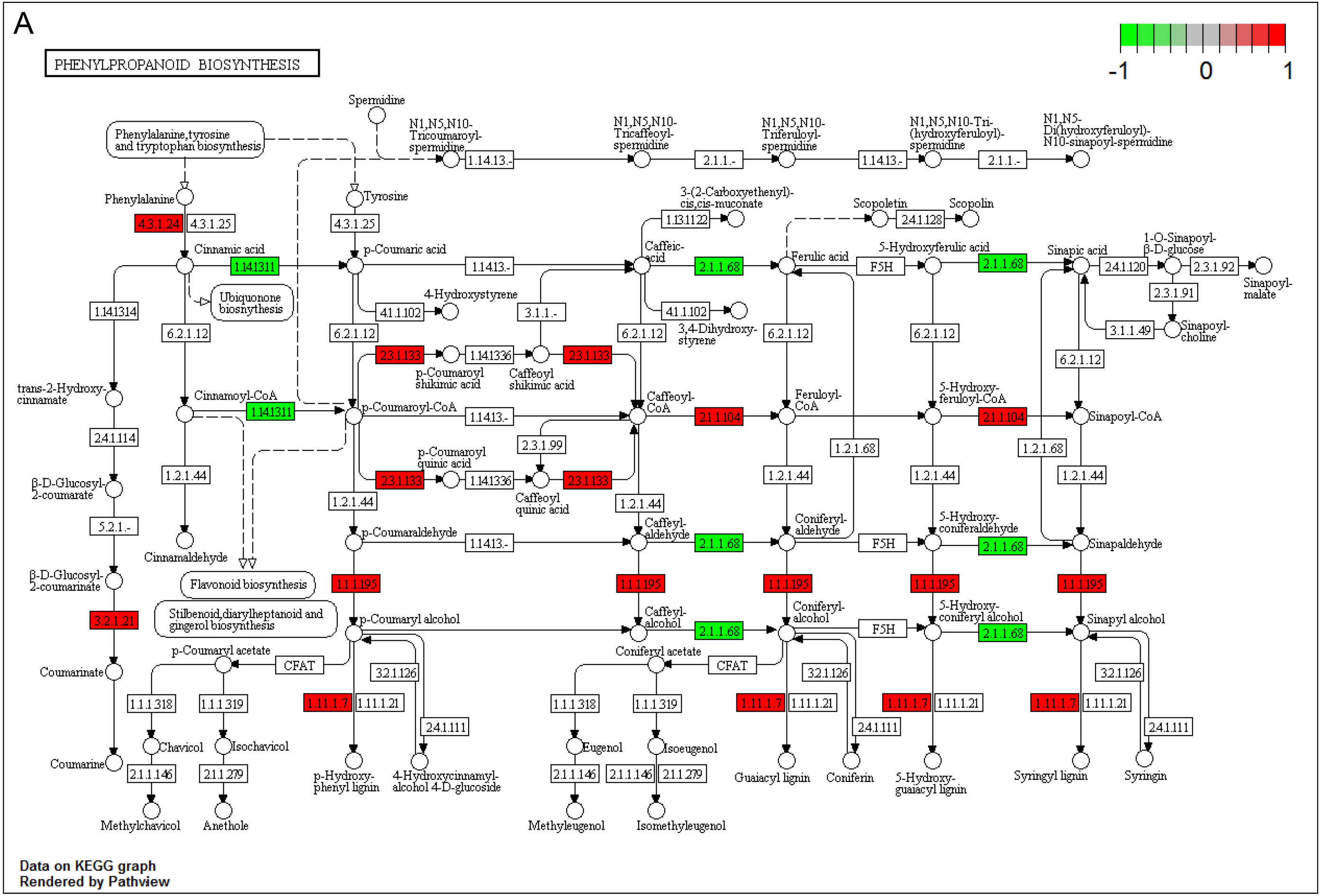

A
PHENYLPROPANOID BIOSYNTHESIS
-1
0
1
Spermidine
N1,N5,N10-Tricoumaroyl-spermidine
1.14.13.-
N1,N5,N10-Tricaffeoyl-spermidine
2.1.1.-
N1,N5,N10-Triferuloyl-spermidine
1.14.13.-
N1,N5,N10-Tri-(hydroxyferuloyl)-spermidine
2.1.1.-
N1,N5-Di(hydroxyferuloyl)-N10-sinapoyl-spermidine
Phenylalanine, tyrosine and tryptophan biosynthesis
Phenylalanine
4.3.1.24
4.3.1.25
Tyrosine
4.3.1.25
3-(2-Carboxyethenyl)-cis,cis-muconate
1.13.11.22
Scopoletin
2.4.1.128
Scopolin
Cinnamic acid
1.14.13.11
p-Coumaric acid
1.14.13.-
Caffeic-acid
2.1.1.68
Ferulic acid
F5H
5-Hydroxyferulic acid
2.1.1.68
Sinapic acid
2.4.1.120
1-O-Sinapoyl-β-D-glucose
2.3.1.92
Sinapoyl-malate
2.3.1.91
3.1.1.49
Sinapoyl-choline
1.14.13.14
Ubiquinone biosynthesis
4.1.1.102
4-Hydroxystyrene
3.1.1.-
4.1.1.102
3,4-Dihydroxy-styrene
6.2.1.12
6.2.1.12
6.2.1.12
6.2.1.12
6.2.1.12
6.2.1.12
2.3.1.133
p-Coumaroyl shikimic acid
1.14.13.36
Caffeoyl shikimic acid
2.3.1.133
trans-2-Hydroxy-cinnamate
Cinnamoyl-CoA
1.14.13.11
p-Coumaroyl-CoA
1.14.13.-
Caffeoyl-CoA
2.1.1.104
Feruloyl-CoA
5-Hydroxy-feruloyl-CoA
2.1.1.104
Sinapoyl-CoA
2.4.1.114
2.3.1.99
1.2.1.68
1.2.1.68
p-Coumaroyl quinic acid
2.3.1.133
1.14.13.36
Caffeoyl quinic acid
2.3.1.133
1.2.1.44
1.2.1.44
1.2.1.44
1.2.1.44
1.2.1.44
1.2.1.44
β-D-Glucosyl-2-coumarate
5.2.1.-
Cinnamaldehyde
p-Coumaraldehyde
1.14.13.-
Caffeyl-aldehyde
2.1.1.68
Coniferyl-aldehyde
F5H
5-Hydroxy-coniferaldehyde
2.1.1.68
Sinapaldehyde
β-D-Glucosyl-2-coumarinate
Flavonoid biosynthesis
1.1.1.195
1.1.1.195
1.1.1.195
1.1.1.195
1.1.1.195
3.2.1.21
Stilbenoid, diarylheptanoid and gingerol biosynthesis
p-Coumaryl alcohol
Caffeyl-alcohol
2.1.1.68
Coniferyl-alcohol
F5H
5-Hydroxy-coniferyl alcohol
2.1.1.68
Sinapyl alcohol
p-Coumaryl acetate
CFAT
Coniferyl acetate
CFAT
Coumarinate
3.2.1.126
3.2.1.126
3.2.1.126
1.1.1.318
1.1.1.319
1.11.1.7
1.11.1.21
2.4.1.111
1.1.1.318
1.1.1.319
1.11.1.7
1.11.1.21
2.4.1.111
1.11.1.7
1.11.1.21
1.11.1.7
1.11.1.21
2.4.1.111
Chavicol
Isochavicol
Eugenol
Isoeugenol
Coumarine
2.1.1.146
2.1.1.279
p-Hydroxy-phenyl lignin
4-Hydroxycinnamyl-alcohol 4-D-glucoside
2.1.1.146
2.1.1.146
2.1.1.279
Guaiacyl lignin
Coniferin
5-Hydroxy-guaiacyl lignin
Syringyl lignin
Syringin
Methylchavicol
Anethole
Methyleugenol
Isomethyleugenol
Data on KEGG graph
Rendered by Pathview

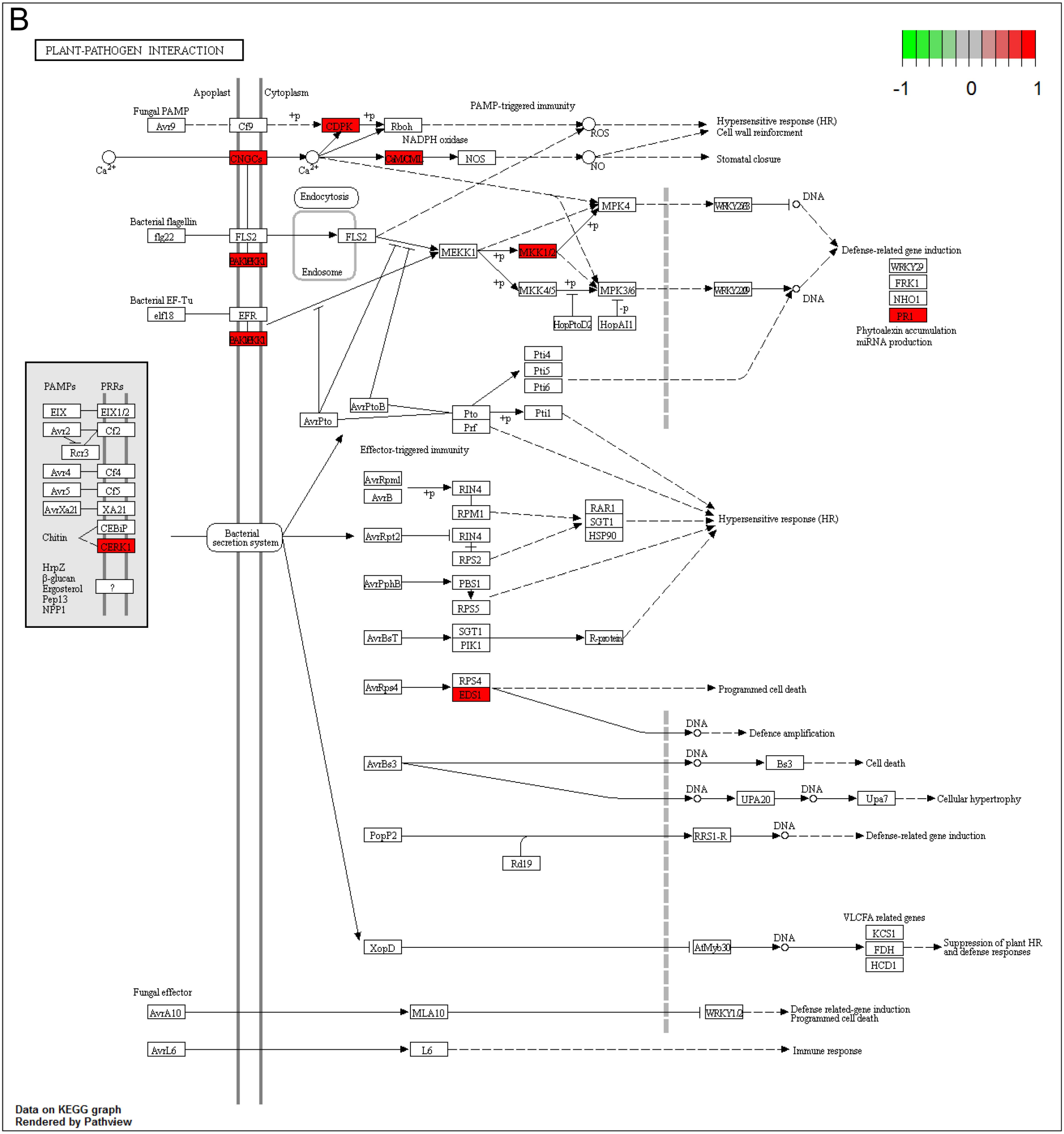

B
PLANT-PATHOGEN INTERACTION
-1
0
1
Apoplast
Cytoplasm
Fungal PAMP
Avr9
Cf9
+p
CDPK
+p
Rboh
NADPH oxidase
PAMP-triggered immunity
ROS
Hypersensitive response (HR)
Cell wall reinforcement
Ca2+
CNGCs
Ca2+
CaM/CML
NOS
NO
Stomatal closure
Endocytosis
Endosome
Bacterial flagellin
flg22
FLS2
FLS2
BAK1/BKK1
MEKK1
MKK1/2
MPK4
WRKY25/33
DNA
Defense-related gene induction
WRKY29
FRK1
NHO1
PR1
Phytoalexin accumulation
miRNA production
MKK4/5
MPK3/6
WRKY22/29
DNA
HopPtoD2
HopAI1
-p
Bacterial EF-Tu
elf18
EFR
BAK1/BKK1
Pti4
Pti5
Pti6
PAMPs
PRRs
EIX
EIX1/2
Avr2
Cf2
Rcr3
Avr4
Cf4
Avr5
Cf5
AvrXa21
XA21
CEBiP
Chitin
CERK1
HrpZ
β-glucan
Ergosterol
Pep13
NPP1
?
AvrPtoB
AvrPto
Pto
Prf
Pti1
Effector-triggered immunity
AvrRpm1
AvrB
RIN4
RPM1
RAR1
SGT1
HSP90
Hypersensitive response (HR)
Bacterial secretion system
AvrRpt2
RIN4
RPS2
AvrPphB
PBS1
RPS5
AvrBsT
SGT1
PIK1
R-protein
AvrRps4
RPS4
EDS1
Programmed cell death
DNA
Defence amplification
AvrBs3
DNA
Bs3
Cell death
DNA
UPA20
DNA
Upa7
Cellular hypertrophy
PopP2
RRS1-R
DNA
Defense-related gene induction
Rd19
VLCFA related genes
XopD
AtMyb30
DNA
KCS1
FDH
HCD1
Suppression of plant HR and defense responses
Fungal effector
AvrA10
MLA10
WRKY1/2
Defense related-gene induction
Programmed cell death
AvrL6
L6
Immune response
Data on KEGG graph
Rendered by Pathview

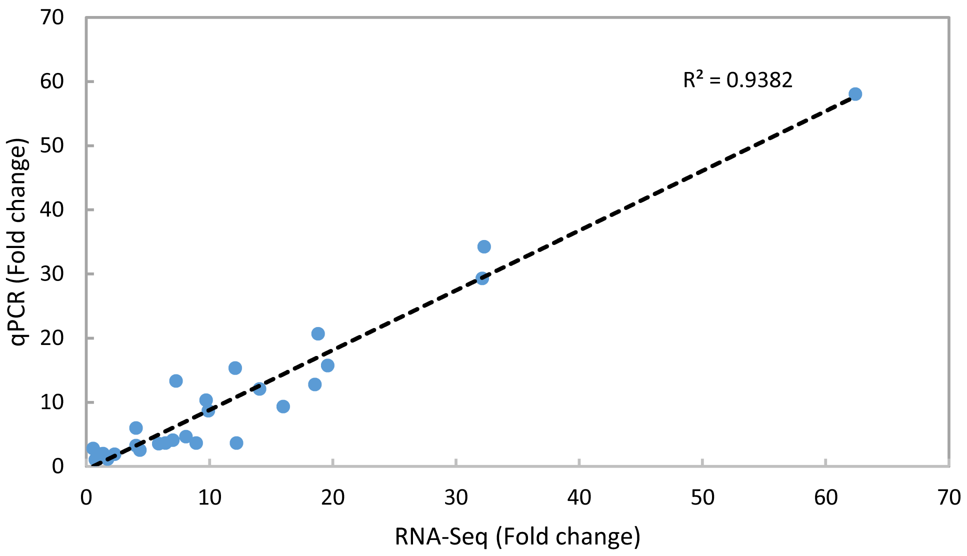

R² = 0.9382
qPCR (Fold change)
RNA-Seq (Fold change)
0
10
20
30
40
50
60
70

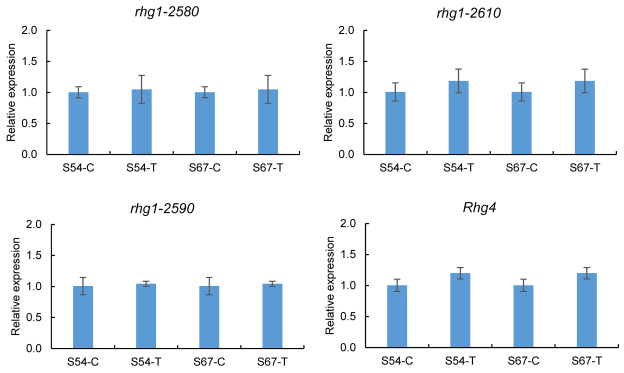

rhg1-2580
rhg1-2610
rhg1-2590
Rhg4
Relative expression
2.0
1.5
1.0
0.5
0.0
S54-C
S54-T
S67-C
S67-T
